# Synthesis and Biological Activity of 3-(Heteroaryl)quinolin-2(1*H*)-ones Bis-Heterocycles as Potential Inhibitors of the Protein Folding Machinery Hsp90

**DOI:** 10.3390/molecules27020412

**Published:** 2022-01-09

**Authors:** Enrique L. Larghi, Alexandre Bruneau, Félix Sauvage, Mouad Alami, Juliette Vergnaud-Gauduchon, Samir Messaoudi

**Affiliations:** 1 CNRS, BioCIS, Université Paris-Saclay, 92290 Châtenay-Malabry, France; mouad.alami@u-psud.fr; 2 Instituto de Química Rosario (IQUIR) CONICET/UNR, FBioyF, Rosario S2002LRK, Argentina; alexandre.bruneau@univesite-paris-saclay.fr; 3 CNRS, Institut Galien-Paris Saclay, Université Paris-Saclay, 92296 Châtenay-Malabry, France; felix.sauvage@ugent.be (F.S.); juliette.vergnaud@universite-paris-saclay.fr (J.V.-G.)

**Keywords:** Hsp90, 6BrCaQ, 3-(heteroaryl)quinolin-2(1*H*)-ones, purines, cytotoxicity

## Abstract

In the context of our SAR study concerning 6BrCaQ analogues as C-terminal Hsp90 inhibitors, we designed and synthesized a novel series of 3-(heteroaryl)quinolin-2(1*H*), of types **3**, **4**, and **5**, as a novel class of analogues. A Pd-catalyzed Liebeskind–Srogl cross-coupling was developed as a convenient approach for easy access to complex purine architectures. This series of analogues showed a promising biological effect against MDA-MB231 and PC-3 cancer cell lines. This study led to the identification of the best compounds, **3b** (IC_50_ = 28 µM) and **4e**, which induce a significant decrease of CDK-1 client protein and stabilize the levels of Hsp90 and Hsp70 without triggering the HSR response.

## 1. Introduction

The 90-kDa heat shock protein (Hsp90) has emerged recently as a promising therapeutic target for the treatment of cancer [1,2,3,4,5] and other diseases [6,7]. As a chaperone protein, Hsp90 is evolved in the conformational maturation, folding, stabilization, activation, and degradation of over 400 client proteins in healthy cells as well as in cancerous cells which are directly associated with all hallmarks of cancer [8,9,10,11]. This Hsp90 chaperone cycle depends on the ATPase activity. ATP binding to the *N*-terminal domain (NTD) and hydrolysis by Hsp90 drive a conformational cycle necessary for chaperone function [12,13,14]. The binding of ATP to each monomer shifts Hsp90 to a “closed” formation that can bind, fold, and activate client proteins [15,16]. Thus, inhibition of Hsp90 function results in the simultaneous interruption of many signal transduction pathways which are pivotal to tumor progression and survival.

Several structurally distinct Hsp90 inhibitors that target the ATP binding pocket are currently being evaluated for anticancer activity in numerous Phase II and several Phase III clinical trials. However, they are ineffective over time due to the compensatory mechanism involving the induction of a heat shock response. The expression of chaperones Hsp27, Hsp70, Hsp40, and Hsp90 increases [17,18,19], leading to undesirable chemoprotective effects [20,21,22]. Clinical resistance has been attributed to this chemoprotective effect, and dosage increases to overcome resistance are not a viable option due to toxicity. These results continue to motivate the pursuit of alternative strategies for modulating heat shock protein complexes [23,24,25].

**Figure 1:**
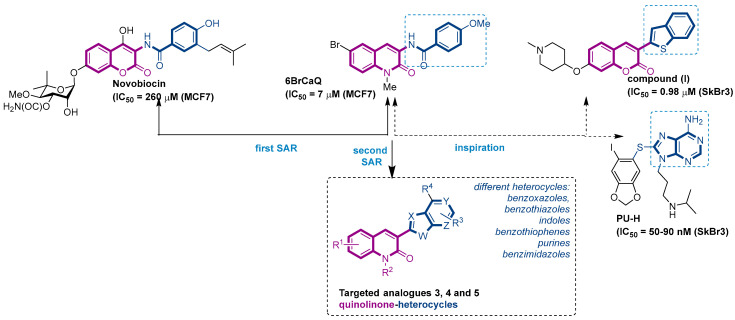
Structure of novobiocin and 6BrCaQ compounds and the approach for the design of targeted compounds **3**, **4** and **5**.

An alternative molecular mechanism of inhibition is through binding to the C-terminal domain of Hsp90. The CTD has been implicated biochemically as the site of a possible second cryptic ATP-binding site on the protein. Its contribution to the overall regulation of chaperone function is not clear, but some small molecules that interact with the C-terminal domain, such as the antibiotic novobiocin [26] (Nvb, Figure 1) and coumermycin A1 (Cm A1), induce client protein degradation without heat shock response induction [27,28,29,30,31], giving new promise to Hsp90 inhibition for cancer treatments.

In this context, we previously reported a novel series of simplified **3** amido-quinolin-2-one analogues related to Nvb as a class of highly potent hsp90 inhibitors [32,33,34,35,36]. From the structure–activity relationship (SAR) studies, 6BrCaQ (Figure 1) [37,38] was identified as a very promising C-terminal Hsp90 inhibitor displaying an antiproliferative activity LC_50_ of 5–50 µM [39,40] against various cancer cell lines (MCF7, MDA MB231, Caco2, IGROV, ISHIKAWA, PC3, and HT29 cells). Further studies on its mode of action revealed that 6BrCaQ manifests downregulation of several Hsp90 client proteins (HER2, Raf-1 and cdk-4), induces a high apoptosis level in MCF-7 breast cancer cell line and PC3. In addition, encapsulated in liposomes, 6BrCaQ exerted an improved in vitro activity on breast cancer cells (MDA-MB-231) and displays an in vivo anti-tumor activity on an orthotopic breast cancer model in nude mice [40].

More recently, we demonstrated that conjugation of 6BrCaQ with the cationic head triphenylphosphonium (TPP) leads to the conjugate 6BrCaQ-C10-TPP for the targeting of the mitochondrial heat shock protein TRAP1. Hence, 6BrCaQ-C10-TPPdisplays an anti-proliferative activity with mean GI_50_ values at a nanomolar level in a diverse set of human cancer cells (GI_50_ = 0.008–0.30 µM) including MDA-MB-231, HT-29, HCT116, K562 and PC-3 cancer cell lines. This study showed that this compound 6BrCaQ-C_10_-TPP induces a significant mitochondrial membrane disruption and interferes with TRAP1 function in colon carcinoma cells without inducing the heat-shock response HSF1 [41].

On the other hand, Blagg and co-workers reported during their various SAR studies that the coumarin analogue (I) (Figure 1), which possesses a benzothiophen heterocycle at the C3 position, is able to induce the cell death with an IC_50_ of 0.98 µM against SkBr3 cell lines [42]. Inspired by this study and the promising activity displayed by 6BrCaQ, we proposed to design a news series of quinolinone based heterocycle analogues (Figure 1) in which the amide function of 6BrCaQ will be replaced by various heterocycles, including benzoxazoles, benzothiazoles, indoles, benzimidazoles, and purines, in the aim to better understand the SAR in this novel series. In this article, the synthesis and biological evaluation of analogues of type **3**, **4,** and **5** are described.

## 2. Results and Discussion

### 2.1. Chemistry

**Scheme 1:**
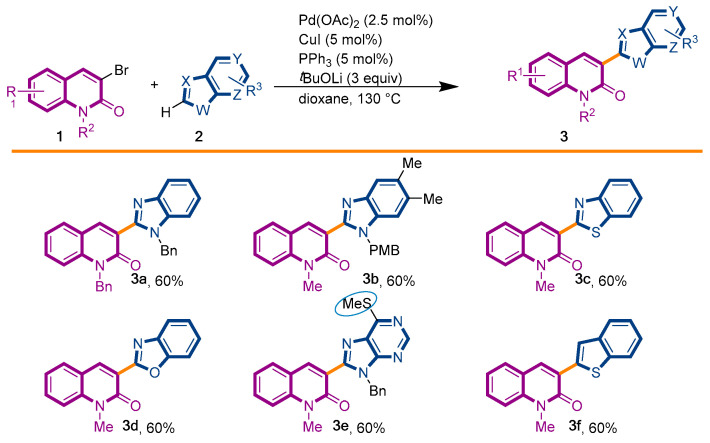
Synthetic strategy to target 3-(*heteroaryl*)quinolin-2(1*H*)-ones **3**.

The first library of compounds targeted is simplified 3-(heteroaryl)quinolin-2(1*H*)-ones **3** (Scheme 1). These analogues were synthesized by the palladium-catalyzed C-H functionalization reaction of 3-bromoquinolin-2-(1*H*)-ones **1** with various azoles according to our previously reported conditions [43]. The reactions take place rapidly in 1,4-dioxane and proceed in good to excellent yields using bimetallic Pd(OAc)_2_/CuI as catalysts, PPh_3_ as the ligand, and LiO*t*Bu as the base. Under this convergent protocol, compounds **3a–f** were synthesized. These compounds were already reported in reference [43], fully characterized, and their physical properties can also be found in ref [43]. Various heterocycles could be introduced at the C3-position of the quinolin-2(1*H*)-one nucleus, including benzoxazole, benzothiazole, indole, benzimidazole, and the SMe-purine (Scheme 1).

In the pursuit of our SAR-study, we considered the possibility of functionalization of the thiomethyl group attached to 6′-position of the purine motif in the derivative **3e**. This motif is found in a series of hsp90 inhibitors, such as PU-H71 disclosed in Figure 1. If succeeded, this approach would provide a fast and easy access to a small library of more sophisticated purine-quinolinone analogues. We have rationalized that an additional aromatic group in molecule **1** would modify the intercalation ability due to changes in the planarity and in the extension of conjugation.

After a detailed survey about this topic, only few examples of this derivatization of thiopurines were found in the literature. One of the methodologies available to introduce diversity in this particular position is the scarcely explored Liebeskind–Srogl coupling [44]. This approach exploits the pseudo-halogen character of CH_3_S- group (thio-organyl in general) as partner in a Suzuki-like coupling reaction, involving an arylboronic acid under palladium catalysis in the presence of a copper salt [45,46].

**Scheme 2:**
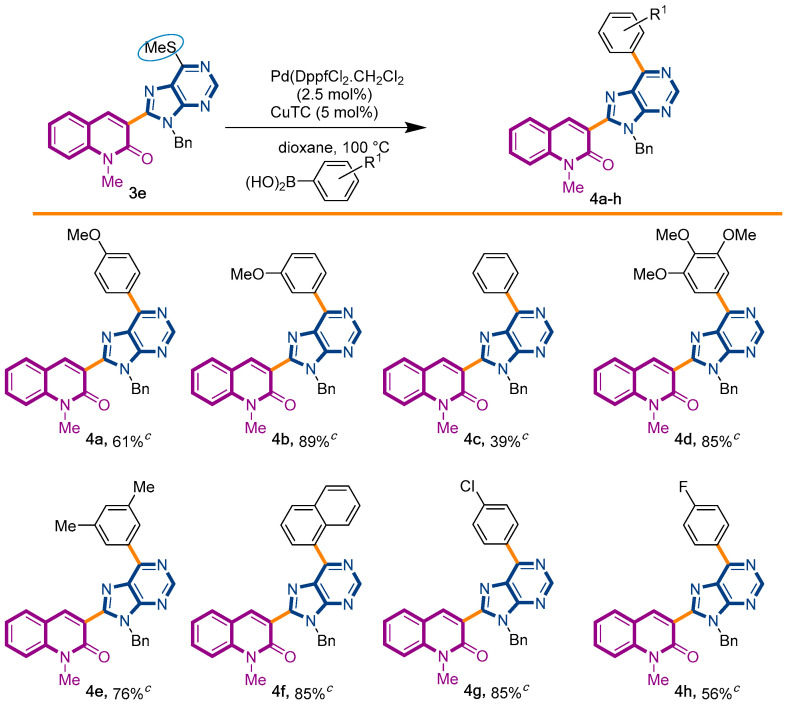
Synthetic strategy to target 3-(purino)-quinolin-2(1*H*)-ones **4a–h**.

We decided to start our investigations with the adoption of two approaches. The first one involved the coupling of thiopurinoquinolone **3e** with *p*-methoxyphenyl-boronic acid under PdDppfCl_2_·CH_2_Cl_2_ catalysis and conventional heating [47], while the second method chosen to promote the desired transformation employed Pd(OAc)_2_ and 1,10-phenanthroline under microwave irradiation [48]. To our delight, both approaches were capable to afford the expected product **4a**, in 61% and 49% yield, respectively (Scheme 2). In order to explore the scope of this synthetic transformation, we decided to use the first method with a sort of arylboronic acid. This selection was based on the rationale of the electronic and steric effects exerted by the chosen substituents. The reactions proceeded smoothly, affording the expected products **4a–h** with yields ranging from 39% to 89% (Scheme 2).

**Scheme 3:**
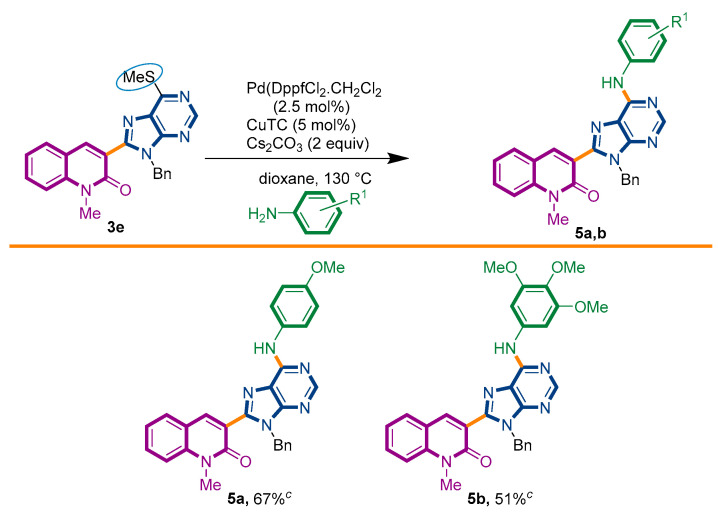
Synthetic strategy to target 3-adenines-quinolin-2(1*H*)-ones **5a**,**b**.

Taking advantage of this synthetic procedure, we decided to examine the scope of Liebeskind–Srogl reaction with anilines (Scheme 3). The introduction of a nitrogen atom at the 6′-position of the purine ring would imply its overall transformation into a nucleic acid analogue, i.e., an [(*N*-phenyl)adenine] motif. The possibility to have an adenine ring attached to a quinolone nucleus would increase its biological resemblance [49,50].

It is interesting to denote that this reaction can also be reached by an alternative two-step procedure involving the initial thioether-sulfone oxidation, followed by a nucleophilic heteroaromatic substitution with the appropriate amine. This protocol has been previously employed by Piguel and coworkers during the synthesis of 6,8,9-purine-derivatives [47]. To the best of our knowledge, the introduction of an amine to the purine ring in 6′-position through a Liebeskind–Srogl reaction has never been reported in the literature.

We started our investigations by adapting the arylsulfide amination protocol described by the group of Murakami [51]. Unfortunately, only the degradation of starting material **3e** was detected. Several conditions were assayed, including the palladium source, ligand, base, and microwave heating [52,53,54]. To our surprise, with a slight modification of the previously used PdDppf·Cl_2_/CuTC protocol, the reaction proceeded until completeness, giving the desired product **5a** in 67% yield (Scheme 3). It is important to observe that the presence of CuTC and the Cs_2_CO_3_ base were mandatory to accomplish the expected transformation.

Under this condition, we succeed to generate the coupling product from 3,4,5-trimethoxyaniline (**5b**, 51%). Unfortunately, however, the reaction of **3e** with aniline, benzyamine, butylamine, and pyrrolidine could not be driven to completeness and the expected product could not be separated from the starting material by current chromatographic purification conditions (CC and preparative TLC).

### 2.2. Biological Evaluation of Quinolones Analogues

#### Antiproliferative Activity

Upon completion of their syntheses, the in vitro activity of quinolone derivatives **3a–f**, **4a–h**, and **5a**,**b** was evaluated by their growth-inhibitory potency in three cancer cell lines. At first, the viability of the synthesized compounds was examined with the MDA-MB-231 MCF-7 breast cancer cell line at concentrations of 10 µM, 15 µM, and 25 µM. Prostate cancer PC-3 cells and human fetal lung fibroblast MRC-5 cell lines were also subjected to this series of compounds at a unique concentration of 15 µM. The quantification of cell survival in these cell lines was established using MTS assays after 72 h exposure (Table 1), and GI_50_ values were estimated at the concentration required to produce 50% inhibition (Table 2).

As shown in Table 1, all these series of analogues induced a significant decrease of the cell viability in MDA-MB-231 cells in a concentration depend manner. At 10 µM concentration the viability percentage of MDA-MB-231 cells decreased until less than 47% under **3a** and **5a** exposure (Table 1). In addition, increasing the concentration at 25 µM, analogues **3a**, **3b**, **4g**, and **4h** importantly affect the growth of MDA-MB-231 cells (~30% survival), clearly demonstrating the bioactivity potential of these compounds.

Then, the cytotoxicity activity was examined with two other cancer cell lines: PC-3 cells and human fetal lung fibroblast MRC-5. As shown in Table 1, almost all the reported compounds do not present any effect against MRC-5 cell lines (>82% survival) at 15 µM concentration, except compounds **4d**, **4e**, and **4g**, which induce a slight effect on the growth of MRC-5 cells (71% to 79% survival). In contrast, PC-3 cells seem to be more sensitive to these derivatives than MRC-5 cells, as we can see in Table 1. Upon exposure of these cell lines at 15 µM concentration, compounds **3b**, **3h**, **3e**, and **4e** were able to decrease the cell viability in PC-3 cells until 56%.

Then, the growth inhibitory activities against PC-3 prostate cancer cell line were measured for the selected 3-heteroaryl-quinolin-2(1*H*)-one derivatives **3a–e**. All the compounds shown in Table 2 display an estimated GI_50_ ranging between 28 and 48 µM. Of the selected derivatives, **3b** showed a significant ability to inhibit cell growth and was the most cytotoxic (GI_50_ = 28 µM) against the PC-3 prostate cancer cell lines.

To provide additional evidence of the growth inhibitory activity manifested by the derivatives, the most active compounds **3a–e**, **4e,** and **5b** were evaluated for their ability to induce the degradation of Hsp90-dependent client protein Cdk4, the most widely studied molecular signature indicative of Hsp90 blockade.

**Figure 2:**
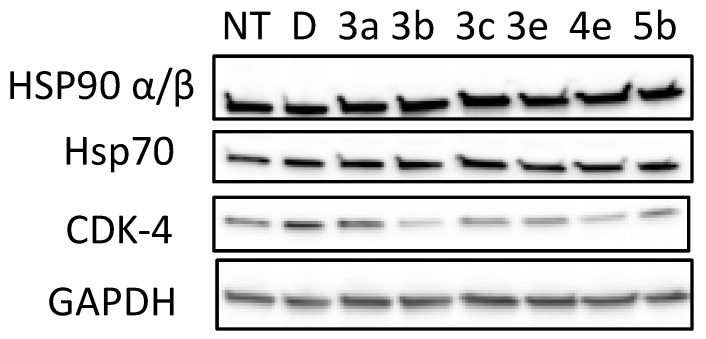
Effects of quinolone analogues **3a–e**, **4e** and **5b** on HSP90 machinery protein levels and on CDK-4 stability. PC-3 cells were grown and exposed to Hsp90 inhibitors (**3a–e**, **4e** and **5b**, 15 µM) as described in Experimental section for 72 h and cell lysates were analyzed by Western blotting with regard to the levels of CDK-4, Hsp90α/β and Hsp70. NT corresponds to untreated cells; D, DMSO-treated cells were used as controls, GADPH level is used for control in protein loading on gels.

As depicted in Figure 2, the cyclin-dependant kinase CdK4 was degraded following treatment with **3a–e**, **4e**, and **5b**. The GAPDH protein was not affected by the tested compounds, indicating the selective degradation of hsp90-dependent clients. CDK-4 level was more decreased by compounds **3b** and **4e** at a concentration of 15 μM. One can note that the anti-proliferative activity of **3b** (IC_50_ = 28 µM, Table 2) and **4e** correlate well with the concentration needed to induce Hsp90/CDK-4 client protein degradation.

Hsp90 *N*-terminal inhibitors induce a Heat-shock-response by releasing a transcription factor (HSF1) of the genes of Hsp27, Hsp70, and Hsp90. This increase in transcription leads to opposition, to apoptosis, and thus resistance to treatment.

It is important to check that the levels of these proteins are not increasing with our compounds. We showed by Western blot that **3a–e**, **4e**, and **5b** stabilize the levels of Hsp90 and Hsp70 without triggering the HSR. This result was already observed with 6-BrCaQ in PC-3 cell lines, as we reported previously: liposomal 6-BrCaQ stabilized levels of Hsp70 and decreased the level of Hsp90 [40].

## 3. Conclusions

In summary, we have designed and synthesized a new series of 3-heteroaryl-quinolin-2(1*H*)-one derivatives as potential Hsp90 inhibitors. During this study, we developed a Pd-catalyzed Liebeskind–Srogl cross-coupling reaction between an SMe-containing quinolinyl-purine derivative and various aryl boronic acids. We reported also, for the first time, that anilines may be used as nucleophilic partners during this coupling. From these SAR studies, **3a–e**, **4e**, and **5b** were found to display the strongest cell viability effect against MDA-MB 231 and PC-3 cancer cell lines. In addition, compounds **3b** and **4e** were found to be able to induce a significant decrease of CDK-1 client protein and stabilize the levels of Hsp90 and Hsp70 without triggering the HSR response.

## 4. Materials and Methods

### 4.1. General Experimental Methods

The compounds were all identified by the usual physical methods, namely ^1^H-NMR, ^13^C-NMR, IR, and HRMS (ESI) (See supplementary materials). The ^1^H and ^13^C NMR spectroscopic data were recorded on a Bruker Avance 300 FT-NMR (300.13 MHz for ^1^H NMR and 75.48 MHz for ^13^C NMR). ^1^H and ^13^C NMR spectra were measured in CDCl_3_ unless otherwise stated. ^1^H chemical shifts are reported in ppm using tetramethylsilane (TMS) as internal standard. The following abbreviations are used: m (multiplet), s (singlet), bs (broad singlet), d (doublet), t (triplet), dd (doublet of doublet), td (triplet of doublet), q (quadruplet), qui (quintuplet), sex (sextuplet). ^13^C NMR chemical shifts are reported in ppm from the central peak of deuterochloroform (77.1). IR spectra were measured on a Bruker Vector 22 spectrophotometer [neat, ATR] and are reported in wave numbers (cm^−1^). High resolution mass spectra (HRMS) were recorded by direct infusion in a mass spectrometer LCT Premier/XE (Waters).

General Methods: All glassware was oven-dried at 140 °C and all reactions were conducted under dry argon atmosphere. The solvents cyclohexane, ethyl acetate and MeOH for chromatography were purchased to Aldrich and were used as received. The reactions were monitored by TLC run in cyclohexane/EtOAc mixtures. The plates were visualized by UV light (254 and 365 nm) and immersed into a solution of phosphomolybdic acid in ethanol, and carefully heated to improve selectivity. Preparative TLC was performed on 2.0-mm thick Merck pre-coated silica gel PLC plates. Merck silica gel 60 (230–400 mesh) was used for column chromatography, employing cyclohexane/EtOAc polarity gradient techniques under positive pressure. Melting points were recorded on a Büchi B-450 apparatus and are uncorrected.

### 4.2. General Procedure for the Liebeskind–Srogl Coupling of 3-(6-Methylthiopurine)-2-quinolone (***3e***) and Boronic Acids

In a reaction tube under argon atmosphere, quinolone **3e** (1 equiv.), boronic acid (2.0 equiv.), PdDppfCl_2_·CH_2_Cl_2_ (0.02 equiv.), and CuTC (2 equiv.) were mixed in dry dioxane (10 mL/mmol). The tube was sealed and placed into a pre-heated oil bath at 80–90 °C until reaction completeness (4–8 h) was ascertained by TLC. The volatiles were removed in the rotavapor, and the crude material was purified by column chromatography or preparative TLC.

3-[9-benzyl-6-(4-methoxyphenyl)-9*H*-purin-8-yl]-1-methylquinolin-2(1*H*)-one (**4a**): 27 mg, 0.056 mmol, Yield 61%. Colorless oil. R_f_ = 0.67 (C_6_H_12_/AcOEt 50%). IR (ATR-diamond, ν): 2963, 1649, 1642, 1598, 1578, 1512, 1453, 1413, 1322, 1292, 1251, 1174, 1103, 1028, 1013, 873, 846, 804, 750, 734, 699 cm^−1^. ^1^H NMR δ: 3.84 (s, 3H, NCH_3_), 3.90 (s, 3H, ArOCH_3_), 5.74 (s, 2H, NCH_2_Ph), 7.00–7.12 (m, 7H, Bn, ArOCH_3_), 7.28 (dt, *J* = 7.9, 0.7 Hz, 1H), 7.42 (d, *J* = 8.7 Hz, 1H), 7.57 (dd, *J* = 7.9, 1.5 Hz, 1H), 7.66 (dd, *J* = 8.3, 0.7 Hz, 1H), 8.05 (s, 1H, H-4), 8.87 (d, *J* = 9.0 Hz, 2H, ArOCH_3_), 9.01 (s, 1H, H-4′). ^13^C NMR δ: 30.1, 47.1, 55.4, 113.5, 114.1, 114.3, 119.7, 122.8, 123.2, 127.4, 128.3, 128.5, 129.9, 130.3, 131.7, 132.4, 136.4, 140.6, 143.2, 151.9, 152.3, 153.8, 154.1, 160.1, 162.0. HRMS *m/z* calcd for C_29_H_25_N_6_O_2_: 489.2039 [M + H]^+^, found: 489.2030.

3-[9-benzyl-6-(3-methoxyphenyl)-9*H*-purin-8-yl]-1-methylquinolin-2(1*H*)-one (**4b**): 47 mg, 0.099 mmol, Yield 89%. Colorless oil. R_f_ = 0.29 (C_6_H_12_/AcOEt 50%). IR (ATR-diamond, ν): 3250, 1646, 1461, 1323, 1218, 1161, 1071, 954, 791 cm^−1^. ^1^H NMR δ (*d*_6_-acetone): 3.87 (s, 3H, NCH_3_), 3.91 (s, 3H, OCH_3_), 5.76 (s, 2H, NCH_2_Ph), 7.07–7.15 (m, 6H, Bn, ArOCH_3_), 7.35 (dt, *J* = 7.9, 0.9 Hz, 1H), 7.49 (d, *J* = 7.9 Hz, 1H), 7.66 (d, *J* = 8.3 Hz, 1H), 7.77 (dt, *J* = 7.9, 1.5 Hz, 1H), 7.81 (d, *J* = 7.9 Hz, 1H), 8.29 (s, 1H), 8.64–8.67 (m, 2H, ArOCH_3_), 9.01 (s, 1H, H-4′). ^13^C NMR δ (*d*_6_-acetone): 30.2, 47.6, 55.7, 115.7, 117.5, 120.5, 123.2, 123.5, 128.3, 128.4, 129.3, 130.3, 130.8, 133.3, 143.9, 153.7, 160.6, 160.8. HRMS *m/z* calcd for C_29_H_24_N_5_O_2_: 474.1930 [M + H]^+^, found: 474.1937.

3-(9-benzyl-6-phenyl-9*H*-purin-8-yl)-1-methylquinolin-2(1*H*)-one (**4c**): 10 mg, 0.024 mmol, Yield 39%. Colorless oil. R_f_ = 0.52 (C_6_H_12_/AcOEt 50%). IR (ATR-diamond, ν): 3060, 2931, 1641, 1586, 1466, 1331, 1298, 1166, 954, 768, 668 cm^−1^. ^1^H NMR δ: 3.84 (s, 3H, NCH_3_), 5.74 (s, 2H, NCH_2_Ph), 7.00–7.13 (m, 5H, Bn), 7.29 (t, *J* = 8.1 Hz, 1H), 7.43 (d, *J* = 8.7 Hz, 1H), 7.48–7.62 (m, 8H), 7.67 (dd, *J* = 8.7, 1.5 Hz, 2H), 8.05 (s, 1H, H-4), 8.25 (d, *J* = 6.8 Hz, 1H, H-4″), 8.81 (dd, *J* = 8.3, 1.9 Hz, 2H, H-2″,6″), 9.08 (s, 1H, H-4′). ^13^C NMR δ: 30.1, 47.1, 114.3, 119.7, 122.9, 123.1, 127.4, 127.7, 127.8, 128.0, 128.5, 128.7, 129.9, 130.7, 130.9, 132.4, 132.7, 133.2, 133.5, 135.9, 136.4, 140.6, 143.3, 152.2, 152.5, 154.3, 154.4, 160.1. HRMS *m/z* calcd for C_28_H_21_N_5_ONa: 466.1644 [M + Na]^+^, found: 466.1646.

3-[9-benzyl-6-(3,4,5-trimethoxyphenyl)-9*H*-purin-8-yl]-1-methylquinolin-2(1*H*)-one (**4d**): 24 mg, 0.053 mmol, Yield 85%. Colorless oil. R_f_ = 0.28 (C_6_H_12_/AcOEt 50%). IR (ATR-diamond, ν): 2939, 2836, 1641, 1505, 1444, 1346, 1217, 11,241,072, 1002, 952, 858, 723, 697 cm^−1^. ^1^H NMR δ: 3.84 (s, 3H, NCH_3_), 3.93 (s, 3H, OCH_3_), 3.938 (s, 6H, OCH_3_), 5.72 (s, 2H, NCH_2_Ph), 6.96–7.02 (m, 2H, Bn), 7.07–7.11 (m, 3H, Bn), 7.28 (t, *J* = 7.4 Hz, 1H), 7.44 (d, *J* = 8.5 Hz, 1H), 7.57 (d, *J* = 7.9 Hz, 1H), 7.68 (dt, *J* = 7.4, 1.5 Hz, 1H) 8.03 (s, 1H), 8.25 (s, 2H, ArOCH_3_), 9.03 (s, 1H, H-4′). ^13^C NMR δ: 30.1, 47.1, 56.3, 61.0, 107.3, 114.4, 119.7, 122.9, 123.4, 127.4, 127.7, 128.5, 129.9, 130.8, 131.3, 132.4, 136.4, 140.6, 143.0, 152.0, 153.3, 153.7, 154.3, 160.1. HRMS *m/z* calcd for C_31_H_28_N_5_O_4_: 534.2141 [M + H]^+^, found: 534.2148.

3-[9-benzyl-6-(3,5-dimethylphenyl)-9*H*-purin-8-yl]-1-methylquinolin-2(1*H*)-one (**4e**): 19 mg, 0.054 mmol, Yield 76%. Colorless oil. R_f_ = 0.61 (C_6_H_12_/AcOEt 50%). IR (ATR-diamond, ν): 3037, 2910, 1646, 1582, 1447, 1322, 1216, 1117, 1073, 955, 863, 725 cm^−1^. ^1^H NMR δ: 3.83 (s, 3H, NCH_3_), 5.73 (s, 2H, NCH_2_Ph), 6.98–7.01 (m, 2H, Bn), 7.07–7.12 (m, 3H, Bn), 7.14 (s, 1H, ArCH_3_), 7.28 (t, *J* = 7.4 Hz, 1H), 7.43 (d, *J* = 8.5 Hz, 1H), 7.59 (d, *J* = 7.7 Hz, 1H), 7.67 (t, *J* = 7.4 Hz, 1H) 8.07 (s, 1H), 8.42 (s, 2H, ArCH_3_), 9.06 (s, 1H, H-4′). ^13^C NMR δ: 21.5, 30.1, 47.1, 114.3, 119.7, 122.8, 123.2, 127.4, 127.6, 127.7, 128.5, 130.0, 130.9, 132.4, 132.7, 135.7, 136.4, 138.2, 140.6, 143.3, 152.1, 152.4, 154.2, 154.8, 160.1. HRMS *m*/*z* calcd for C_30_H_26_N_5_O: 472.2137 [M + H]^+^, found: 472.2139.

3-[9-benzyl-6-(naphthalen-2-yl)-9*H*-purin-8-yl]-1-methylquinolin-2(1*H*)-one (**4f**): 21 mg, 0.059 mmol, Yield 85%. Colorless oil. R_f_ = 0.61 (C_6_H_12_/AcOEt 50%). IR (ATR-diamond, ν): 3067, 1642, 1570, 1446, 1320, 1276, 1168, 920, 847, 754, 696 cm^−1^. ^1^H NMR δ: 3.85 (s, 3H, NCH_3_), 5.76 (s, 2H, NCH_2_Ph), 7.02–7.12 (m, 5H, Bn), 7.29 (t, *J* = 8.1 Hz, 1H), 7.43 (d, *J* = 8.5 Hz, 1H), 7.48–7.56 (m, 2H), 7.60 (d, *J* = 7.3 Hz, 1H), 7.67 (d, *J* = 7.7 Hz, 1H), 7.88 (d, *J* = 8.7 Hz, 1H), 7.99 (d, *J* = 8.7 Hz, 1H), 8.05 (d, *J* = 7.3 Hz, 1H), 8.11 (s, 1H, H-4), 8.97 (d, *J* = 8.3 Hz, 1H, Naphthyl), 9.12 (s, 1H, H-4′), 9.46 (s, 1H, Naphthyl). ^13^C NMR δ: 30.1, 47.2, 114.3, 119.7, 122.9, 123.2, 126.2, 126.3, 127.3, 127.5, 127.70, 127.74, 128.2, 128.5, 123.0, 130.8, 131.2, 132.4, 133.3, 133.4, 133.6, 134.6, 136.4, 140.6, 143.4, 152.3, 152.5, 154.1, 154.4, 160.1. HRMS *m/z* calcd for C_32_H_24_N_5_O: 494.1981 [M + H]^+^, found: 494.1976.

3-[9-benzyl-6-(4-chlorophenyl)-9*H*-purin-8-yl]-1-methylquinolin-2(1*H*)-one (**4g**): 34 mg, 0.083 mmol, Yield 85%. Colorless solid, m.p.: 202–203 °C (CH_2_Cl_2_/MeOH). R_f_ = 0.65 (C_6_H_12_/AcOEt 50%). IR (ATR-diamond, ν): 3044, 2928, 1641, 1582, 1492, 1381, 1298, 1177, 1089, 953, 803, 736, 720 cm^−1^. ^1^H NMR δ: 3.85 (s, 3H, NCH_3_), 5.73 (s, 2H, NCH_2_Ph), 6.99–7.03 (m, 2H, Bn), 7.07–7.12 (m, 3H, Bn), 7.30 (dt, *J* = 7.9, 0.7 Hz, 1H), 7.44 (d, *J* = 8.5 Hz, 1H), 7.51 (d, *J* = 8.5 Hz, 2H, ArCl), 7.69 (dd, *J* = 8.7, 1.7 Hz, 1H), 8.04 (s, 1H), 8.85 (d, *J* = 8.5 Hz, 2H, ArCl), 9.06 (s, 1H, H-4′). ^13^C NMR δ: 30.1, 47.2, 114.3, 119.7, 122.9, 123.0, 127.5, 127.8, 128.9, 130.0, 130.8, 131.2, 132.5, 134.4, 136.3, 137.0, 140.6, 143.3, 152.4, 152.9, 154.4, 160.0. HRMS *m/z* calcd for C_28_H_21_N_5_OCl: 478.1435 [M + H]^+^, found: 478.1440.

3-[9-benzyl-6-(4-fluorophenyl)-9*H*-purin-8-yl]-1-methylquinolin-2(1*H*)-one (**4h**): 22 mg, 0.047 mmol, Yield 56%. Colorless oil. R_f_ = 0.57 (C_6_H_12_/AcOEt 50%). IR (ATR-diamond, ν): 3058, 2958, 1642, 1569, 1463, 1320, 1297, 1159, 1070, 952, 847, 724, 698 cm^−1^. ^1^H NMR δ: 3.63 (s, 3H, NCH_3_), 5.65 (s, 2H, NCH_2_Ph), 6.91–6.94 (m, 2H, Bn), 6.99–7.05 (m, 3H, Bn), 7.14 (t, *J* = 8.9 Hz, 2H, ArF), 7.22 (t, *J* = 7.9 Hz, 1H), 7.36 (d, *J* = 8.5 Hz, 1H), 7.51 (dd, *J* = 7.9, 1.3 Hz, 1H), 7.61 (dt, *J* = 8.5, 1.3 Hz, 1H), 7.97 (s, 1H, H-4), 8.84 (dd, *J* = 8.9, 5.6 Hz, 1H, ArF), 8.97 (s, 1H, H-4′). ^13^C NMR δ: 30.1, 47.1, 67.1 114.3, 115.7 (d, *^2^J_C-F_* = 21.6 Hz), 119.7, 122.9, 123.1, 127.5, 127.8, 128.5, 130.0, 132.1 (d, *^3^J_C-F_* = 8.3 Hz), 136.3, 140.6, 143.3, 152.3, 152.5, 153.0, 154.3, 160.1, 164.5 (d, *^1^J_C-F_* = 249.8 Hz). HRMS *m/z* calcd for C_28_H_20_N_5_OFNa: 484.1550 [M + Na]^+^, found: 484.1552.

### 4.3. Procedure for the Liebeskind–Srogl Coupling of 3-(6-Methylthiopurine)-2-quinolone (***3e***) and Anilines

In a reaction tube equipped with a stirring bar under argon atmosphere, quinolone **3e** (1 equiv.), the appropriate aniline (2.0 equiv.), PdDppfCl_2_·CH_2_Cl_2_ (0.1 equiv.), Ph_3_P (0.2 equiv.), Cs_2_CO_3_ (2.0 equiv.), and CuTC (2 equiv.) were admixed in dry dioxane (20 mL/mmol). The tube was sealed and poured into a pre-heated oil bath at 130 °C until consumption of quinolone **1**, monitored by TLC (15–18 h). The volatiles were removed in the rotavapor, and the crude material was suspended with AcOEt, filtered through a short pad of cotton, concentrated under vacuum, and the remaining solid was purified by preparative TLC (2 × C_6_H_12_/AcOEt 50%).

3-{9-benzyl-6-[(4-methoxyphenyl)amino]-9*H*-purin-8-yl}-1-methylquinolin-2(1*H*)-one (**5a**): 20 mg, 0.039 mmol, Yield 67%. Yellowish oil. R_f_ = 0.36 (2 × C_6_H_12_/AcOEt 50%). IR (ATR-diamond, ν): 3283, 3038, 2928, 2847, 1641, 1588, 1573, 1461, 1380, 1241, 1180, 1087, 953, 829, 755, 697 cm^−1^. ^1^H NMR δ: 3.74 (s, 3H, NCH_3_), 3.75 (s, 3H, OCH_3_), 5.54 (s, 2H, NCH_2_Ph), 6.92 (d, *J* = 8.9 Hz, 2H, Ar), 6.99–7.02 (m, 2H, Bn), 7.09–7.13 (m, 3H, Bn), 7.26 (t, *J* = 8.2 Hz, 1H, H-6), 7.41 (d, *J* = 8.5 Hz, 1H, H-8), 7.48 (d, *J* = 7.7 Hz, 1H, H-5), 7.62–7.68 (m, 3H, H-7, ArOCH_3_), 7.71 (s_b_, 1H, N*H*) 7.86 (s, 1H, H-4), 8.55 (s, 1H, H-4′). ^13^C NMR δ: 30.0, 47.1, 55.5, 114.3, 119.6, 119.9, 122.5, 122.8, 123.0, 127.4, 127.9, 128.5, 129.8, 131.7, 132.2, 136.6, 140.5, 142.4, 147.9, 151.2, 152.2, 153.2, 156.1, 160.1. HRMS *m/z* calcd for C_29_H_25_N_6_O_2_: 489.2039 [M + H]^+^, found: 489.2030.

3-{9-benzyl-6-[(3,4,5-trimethoxyphenyl)amino]-9*H*-purin-8-yl}-1-methylquinolin-2(1*H*)-one (**5b**): 14 mg, 0.025 mmol, Yield 51%. Yellowish oil. R_f_ = 0.18 (3 × C_6_H_12_/AcOEt 50%). IR (ATR-diamond, ν): 3296, 3039, 2933, 2834, 1642, 1588, 1463, 1323, 1126, 1088, 954, 697 cm^−1^. ^1^H NMR δ: 3.82 (s, 3H, NCH_3_), 3.83 (s, 3H, OCH_3_), 3.87 (s, 6H, OCH_3_), 5.63 (s, 2H, N-CH_2_Ph), 6.99–7.02 (m, 2H, Bn), 7.09–7.14 (m, 3H, Bn), 7.16 [s, 2H, Ar(OCH_3_)_3_], 7.27 (t, *J* = 7.4 Hz, 1H, H-6), 7.42 (d, *J* = 8.5 Hz, 1H, H-8), 7.49 (d, *J* = 7.2 Hz, 1H, H-5), 7.66 (t, *J* = 7.9 Hz, 1H, H-7), 7.87 (s_b_, 1H, NH), 8.31 (s, 1H, H-4), 8.59 (s, 1H, H-10). ^13^C NMR δ: 30.1, 47.1, 56.1, 61.0, 98.0, 114.3, 119.6, 119.8, 122.8, 122.8, 127.4, 127.7, 128.5, 129.8, 132.3, 135.1, 136.5, 140.5, 142.5, 148.2, 151.9, 153.1, 153.3, 160.2. HRMS *m/z* calcd for C_31_H_29_N_6_O_4_: 549.2250 [M + H]^+^, found: 549.2250.

### 4.4. Materials and Methods for Cell Culture and Western Blot Analysis

MDA-MB-231 cells were grown in L15 supplemented with 15% serum, 2 mM glutamine, and 22 mM sodium bicarbonate in the presence of Penicillin/Streptomycin antibiotic mixture. PC-3 cells were cultured in RPMI 1640 supplemented with 10% serum and 2 mM glutamine in the presence of Penicillin/Streptomycin antibiotic mixture. MRC-5 cells were cultured in EMEM supplemented with 10% serum and 2 mM glutamine in the presence of Penicillin/Streptomycin antibiotic mixture (Reagents were purchased from Sigma Aldrich, Saint-Quentin-Fallavier, France).

Cells were treated for 72 h with 10, 15, and 25 µM of the selected substances. Control cells were treated with the equivalent in DMSO.

Cell survival was assessed using the CellTiter Aqueous One Proliferation assay (Promega, Charbonnières-les-Bains, France) whereby 2500 or 5000 cells per well were seeded in 96-well plates in 100 µL. At the end of the treatment, 20 µL of reagent was added, and absorbance readings were taken at 492 nm on a 96-well plate reader after 3 h of contact (on average).

For protein expression analysis, cells were seeded at a rate of 0.75 × 10^6^ cells on a 25 cm^2^ surface. At the end of the treatment the cells were washed and lysed with RIPA buffer (Sigma Aldrich, Saint-Quentin-Fallavier, France city, country) to which protease inhibitors (Sigma Aldrich, Saint-Quentin-Fallavier, France SIGMA) were added. After 30 min of lysis on ice, the samples were centrifuged (3500 rpm, 10 min) and stored at −20 °C. Total protein concentration was obtained using the Bio-rad Protein Assay reagent (Bio-Rad Laboratories, Marnes-la-Coquette, France city, country). Hence, 30 µg of protein (denatured in the presence of Laemmli buffer, sample buffer from Bio-Rad) was plated on a 4–15% pre-cast acrylamide gel (Bio-Rad, Marnes-la-Coquette, France) and subjected to SDS-PAGE (150 V, 1 h) and PVDF membrane transfer (100 V, 45 min). Immunorevelation was performed as follows: 1 h saturation of the membrane with TBS-Tween (0.1%)–5% skim milk followed by overnight incubation in primary antibody solution (antibodies were from Santa Cruz Biotechnologies, Clinisciences, Nanterre, France or from Sigma Aldrich, Saint-Quentin-Fallavier, France)(See Table 3). After 1 h of washing in 0.1% TBS-Tween, the membranes were contacted with the second horseradish peroxidase-coupled antibody. Detection of the chemiluminescence signal (SuperSignal Pierce reagent, Fisher Scientific, Illkirch, France) was performed using the ChemiBis gel Imager (DNR Bio-Imaging Systems, Israel).

## Figures and Tables

**Table 1:** Cell viability effect of **3a–f**, **4a–h** and **5a**,**b** derivatives against MDA-MB-231, PC-3 and MRC-5 cell lines measured through cell metabolic activity (MTS-based assay).

	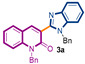			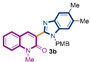			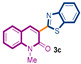			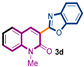			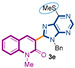			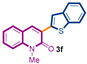		
**Cell viability [%] [a]**																		
**MDA-MB-231**	**10 µM**	**15 µM**	**25 µM**	**10 µM**	**15 µM**	**25 µM**	**10 µM**	**15 µM**	**25 µM**	**10 µM**	**15 µM**	**25 µM**	**10 µM**	**15 µM**	**25 µM**	**10 µM**		
	47	47	36	60	60	23	55	72	65	**98**			73	70	50	**98**		
**PC-3 (15 µM)**	70			61			61			**ND**			61			**ND**		
**MRC-5 (15 µM)**	**80**			**95**			**88**			**ND**			**88**			**ND**		
	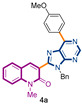			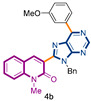			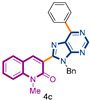			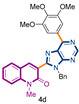			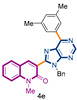			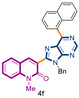		
**Cell viability [%] [a]**																		
**MDA-MB-231**	**10 µM**	**15 µM**	**25 µM**	**10 µM**	**15 µM**	**25 µM**	**10 µM**	**15 µM**	**25 µM**	**10 µM**	**15 µM**	**25 µM**	**10 µM**	**15 µM**	**25 µM**	**10 µM**	**15 µM**	**25 µM**
	66	81	87	70	69	61	71	62	35	60	47	55	57	62	50	73	81	92
**PC-3 (15 µM)**	73			**106**			**97**			**89**			56			**88**		
**MRC-5 (15 µM)**	**94**			**92**			**82**			70			71			**98**		
	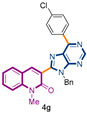			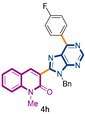			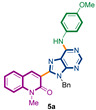			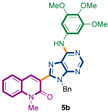			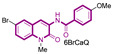			DMSO		
**Cell viability [%] [a]**																		
**MDA-MB-231**	**10 µM**	**15 µM**	**25 µM**	**10 µM**	**15 µM**	**25 µM**	**10 µM**	**15 µM**	**25 µM**	**10 µM**	**15 µM**	**25 µM**	**10 µM**			100		
	56	57	27	55	42	17	62	63	69	44	55	61	76					
**PC-3 (15 µM)**	**91**			**97**			**87**			**88**			ND			100		
**MRC-5 (15 µM)**	79			**84**			**91**			**81**			ND			100		

[a] Value of the anti-proliferative or cytotoxic effect measured by MTS assay (% of viable cells compared to untreated cells 100%) of analogues **3a–f**, **4a–h** and **5a**,**b** derivatives against MDA-MB-231, PC-3 and MCR-5-7 cell lines at the indicated concentrations. ND: not determined. Cell viability: Green means <69%, Orange means 70% to 80%, red means >80%.

**Table 2:** GI_50_ (µM) values for anti-proliferative effects of selected compounds **3a–e** [a].

Compound	PC-3
**6Br-CaQ**	10
**3a**	48
**3b**	28
**3c**	37
**3e**	38

[a] GI_50_ is the concentration of compound needed to reduce cell growth by 50% following 72 h cell treatment with the tested drug.

**Table 3:** Informations concerning antibodies used during this study.

Primary Antibody	Dilution	Secondary Antibody (from Santa-Cruz)	Dilution
Anti-Hsp90 α/β (H-114) (Santa-Cruz)	1/500	Anti-rabbit	1/10,000
Anti-Hsp70 (Santa-Cruz)	1/500	Anti-mouse	1/3000
Anti-CDK-4 (C-22) (Santa-Cruz)	1/500	Anti-rabbit	1/10,000
Anti-GAPDH (Sigma Aldrich)	1/5000	Anti-rabbit	1/10,000

## Data Availability

Data supporting the reported results will be available from the corresponding author (E. L. Larghi, or S. Messaoudi).

## Supplementary Materials

The following are available online, 1*H* and ^13^C NMR spectra for compounds **4a–h** and **5a**, **5b**.

## Sample Availability

Samples of the compounds are available from the authors.
