# Supplementary material for: Synthesis and Biological Activity of 3-(Heteroaryl)quinolin-2(1H)-ones Bis-Heterocycles as Potential Inhibitors of the Protein Folding Machinery Hsp90
Source: Molecules. 2022 Jan 9;27(2):412. doi: 10.3390/molecules27020412 (PMC8778022; doi:10.3390/molecules27020412)
Supplement: Supplementary file 1 [file molecules-27-00412-s001.zip › molecules-1553213-supplementary.pdf]

Supporting Information

Synthesis and Biological Activity of 3-(heteroaryl)quinolin-2(1*H*)-ones bis-Hetereocycles as potential Inhibitors of the Protein Folding Machinery Hsp90

Enrique L. Larghi,[a,b,*] Alexandre Bruneau,[b] Félix Sauvage,[c] Mouad Alami,[b] Juliette Vergnaud- Gauduchon[c] and Samir Messaoudi [a,*]

[a] Université Paris-Saclay, CNRS, BioCIS, 92290, Châtenay-Malabry, France;
[b] Instituto de Química Rosario (IQUIR) CONICET/UNR, FBioyF, Rosario, S2002LRK, Argentina;
[c] Université Paris-Saclay, CNRS, Institut Galien-Paris Saclay, 92296 Châtenay-Malabry, France.
* Correspondence: larghi@iquir-conicet.gov.ar;
samir.messaoudi@universite-paris-saclay.fr

Table of content

**1. ^{1}H and ^{13}C NMR spectra for compounds 4a-h and 5a,b**	2

4a, ^{1}H NMR, $CDCl_3$, 300 MHz

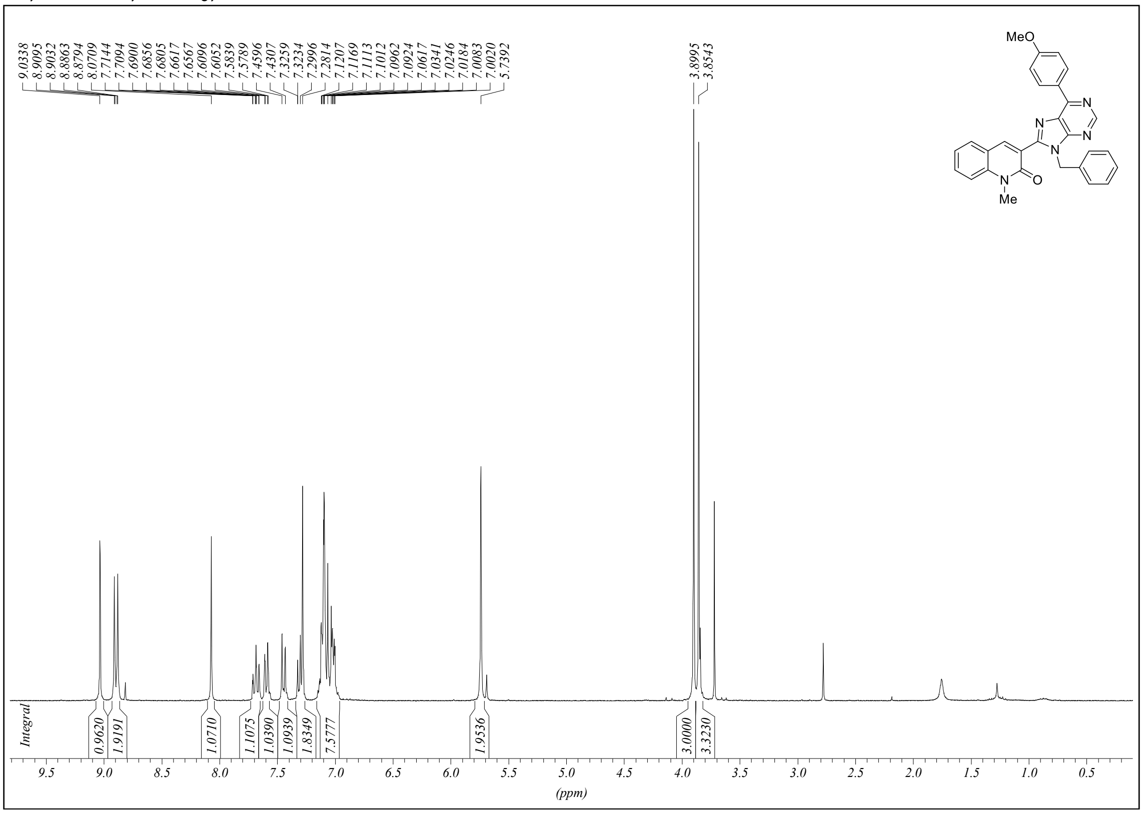

4a, ^{13}C NMR, $CDCl_3$, 75 MHz

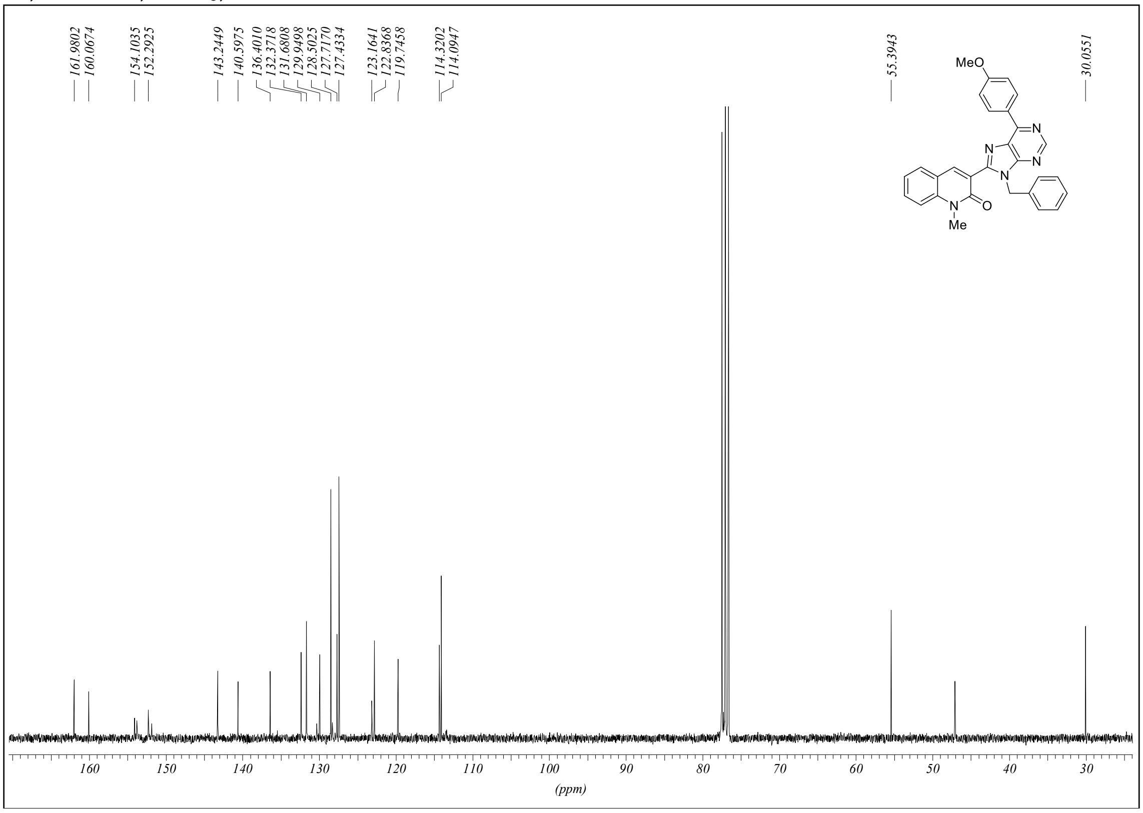

4a, HRMS

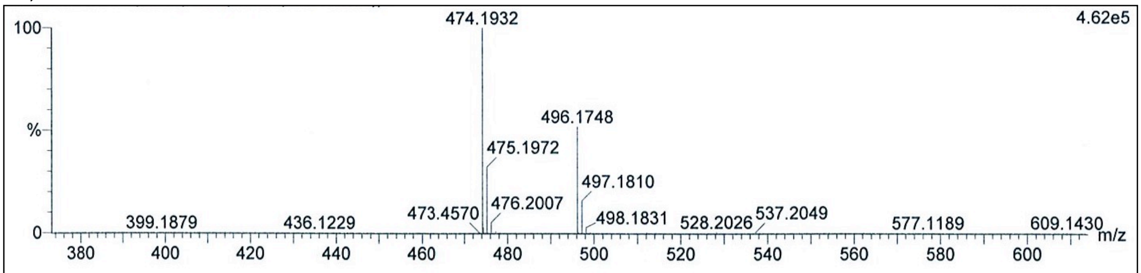

Minimum:	50.00				-2.0		
Maximum:	100.00		200.0	5.0	100.0		
Mass	RA	Calc. Mass	mDa	PPM	DBE	i-FIT	Formula
474.1932	100.00	474.1930	0.2	0.4	20.5	731.8	C29 H24 N5 O2
		474.1946	-1.4	-3.0	21.5	1996.8	C32 H25 N3 Na
		474.1917	1.5	3.2	15.5	589.1	C28 H28 N O6
496.1748	51.81	496.1749	-0.1	-0.2	20.5	662.5	C29 H23 N5 O2 Na
		496.1760	-1.2	-2.4	18.5	934.1	C30 H26 N O6
		496.1736	1.2	2.4	15.5	282.6	C28 H27 N O6 Na

4b, ^{1}H NMR, d_6-acetone, 300 MHz

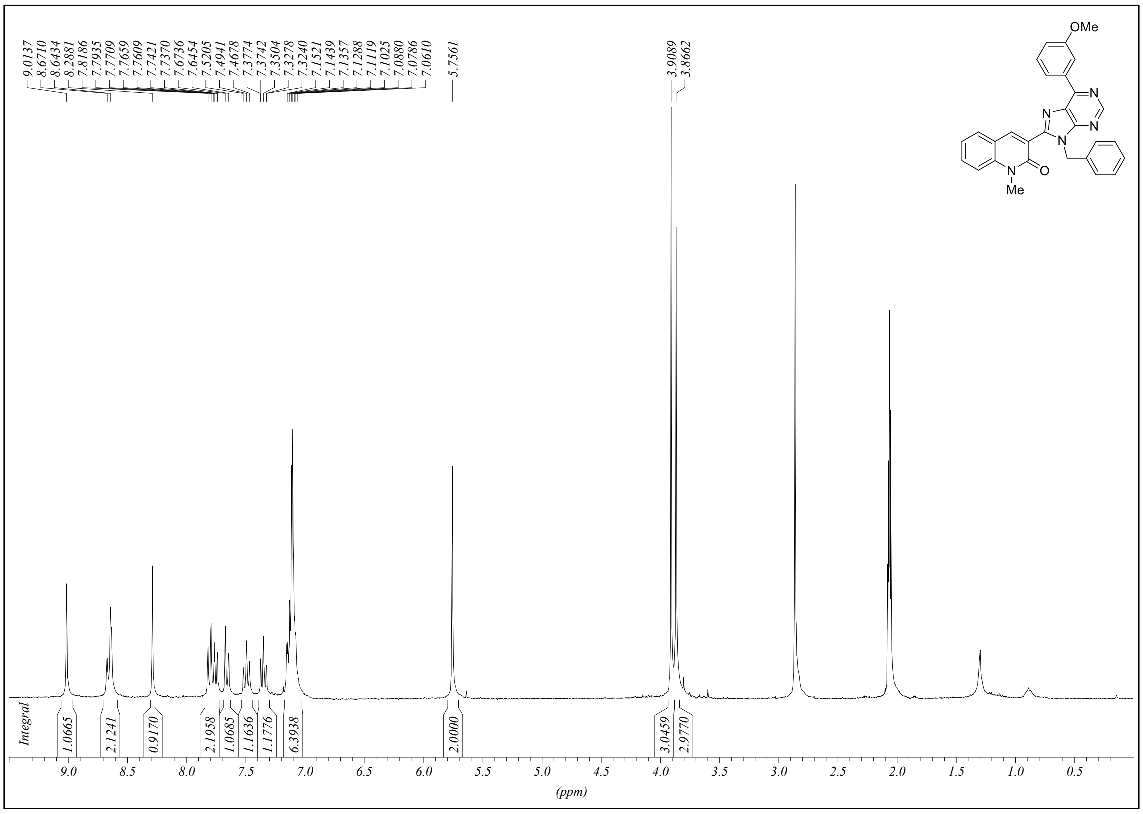

4b, JMOD, d_6-acetone, 75 MHz

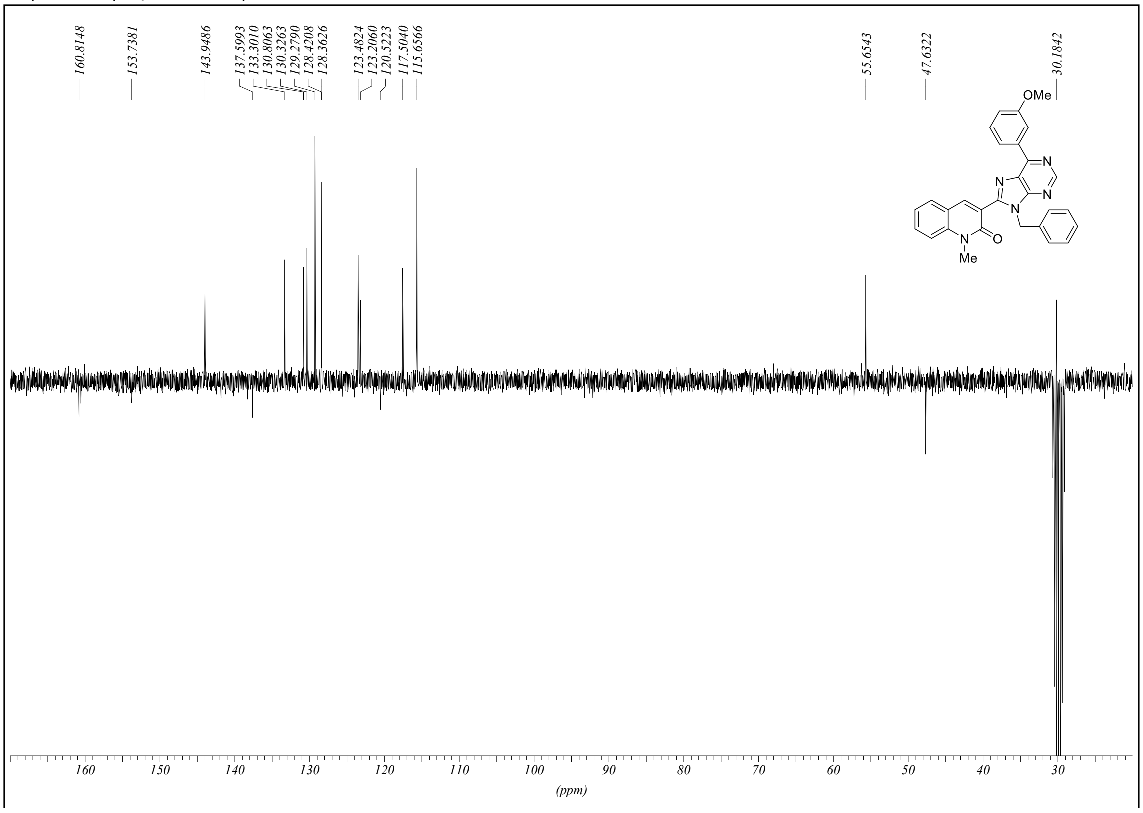

4b - HRMS

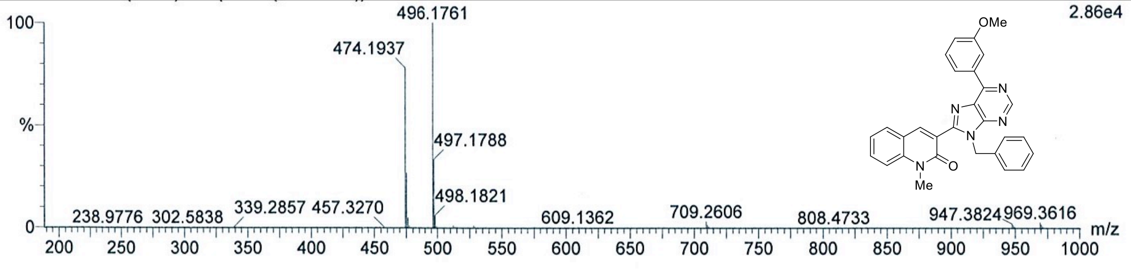

Minimum:	50.00				-2.0		
Maximum:	100.00		200.0	5.0	100.0		
Mass	RA	Calc. Mass	mDa	PPM	DBE	i-FIT	Formula
474.1937	77.95	474.1930	0.7	1.5	20.5	1.2	C29 H24 N5 O2
		474.1917	2.0	4.2	15.5	9.6	C28 H28 N O6
		474.1958	-2.1	-4.4	17.5	4.8	C29 H26 N3 O F Na
496.1761	100.00	496.1760	0.1	0.2	18.5	20.7	C30 H26 N O6
		496.1749	1.2	2.4	20.5	5.4	C29 H23 N5 O2 Na
		496.1785	-2.4	-4.8	19.5	2.8	C28 H23 N5 O3 F

4c, ^{1}H NMR, $CDCl_3$, 300 MHz

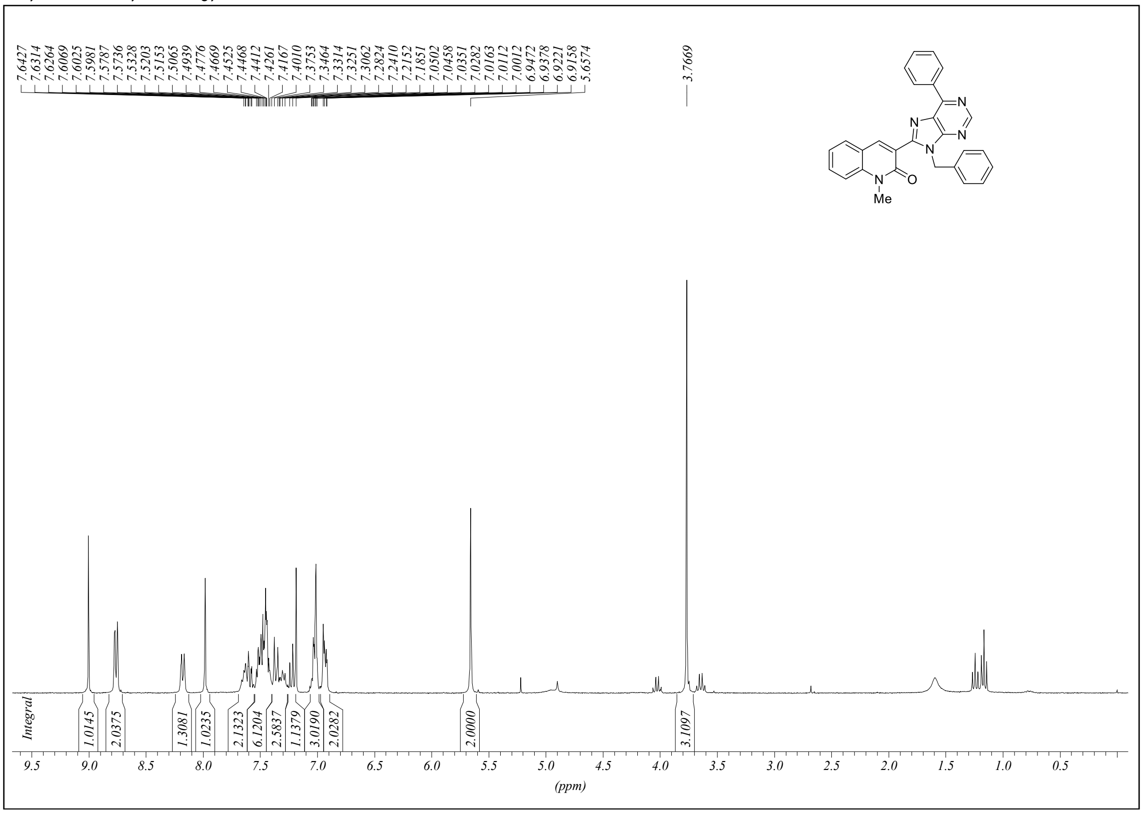

4c, JMOD-NMR, $CDCl_3$, 75 MHz

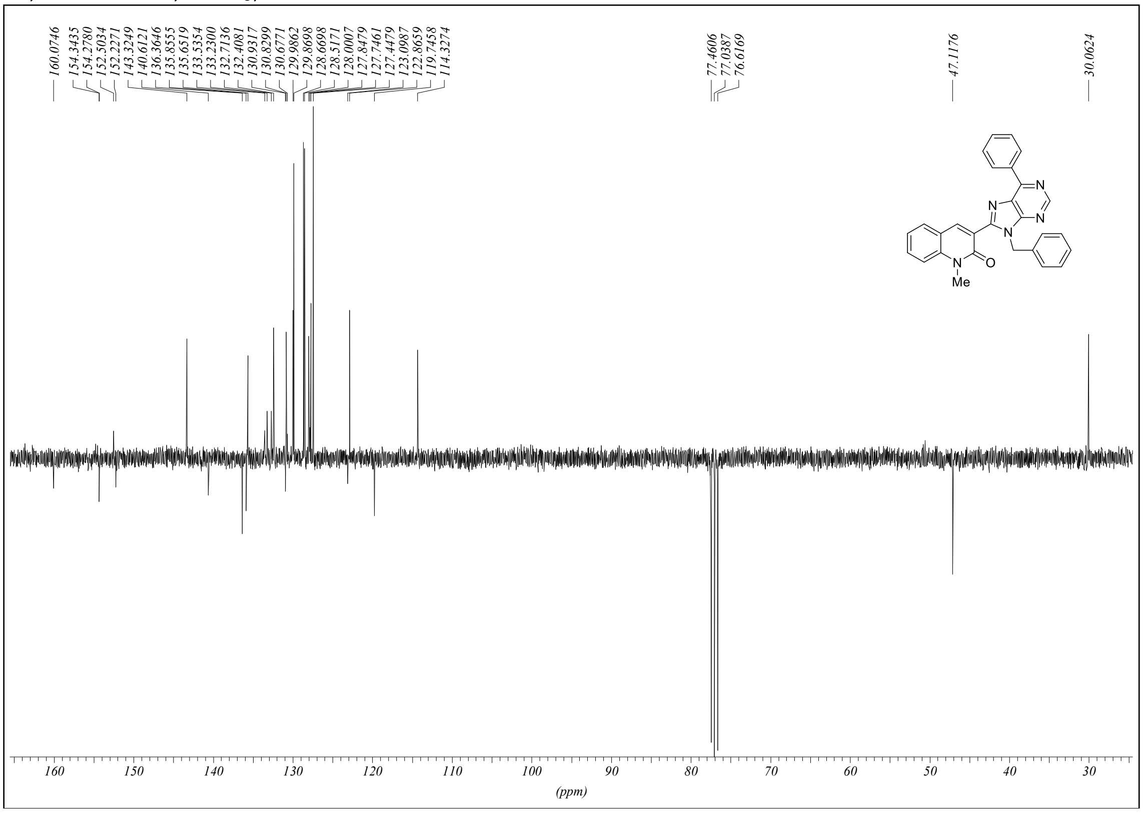

4c - HRMS

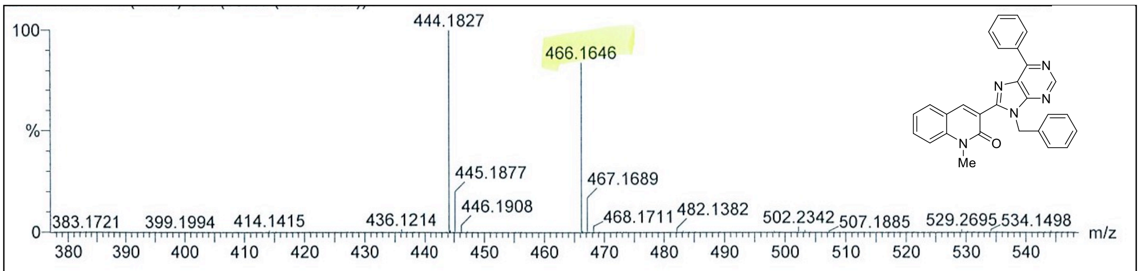

Minimum:	50.00				-2.0		
Maximum:	100.00		200.0	5.0	100.0		
Mass	RA	Calc. Mass	mDa	PPM	DBE	i-FIT	Formula
444.1827	100.00	444.1829	-0.2	-0.5	2.5	592.3	C15 H30 N3 O12
		444.1824	0.3	0.7	20.5	16065.0	C28 H22 N5 O
		444.1819	0.8	1.8	4.5	168.1	C14 H27 N7 O8 Na
466.1646	84.12	466.1644	0.2	0.4	20.5	13446.5	C28 H21 N5 O Na
		466.1649	-0.3	-0.6	2.5	548.9	C15 H29 N3 O12 Na
		466.1654	-0.8	-1.7	18.5	14574.6	C29 H24 N O5

4d, ^1H NMR, $CDCl_3$, 300 MHz

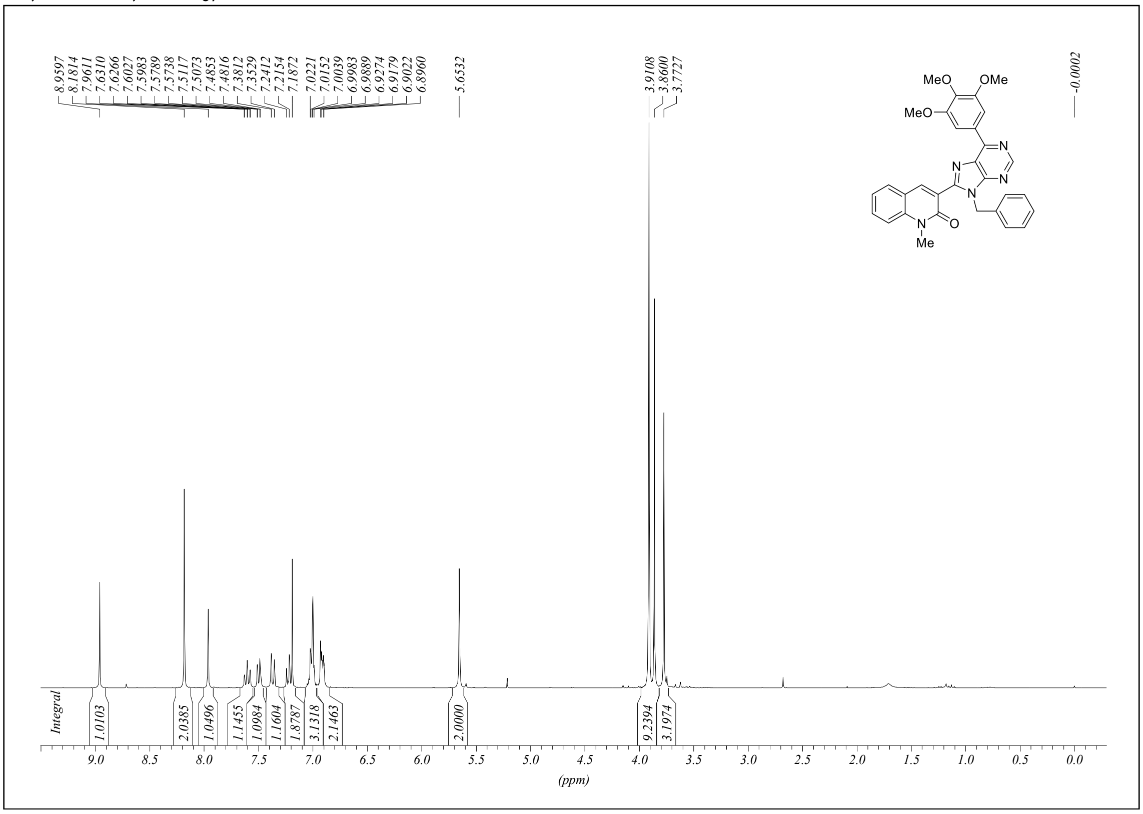

4d, JMOD-NMR, $CDCl_3$, 75 MHz

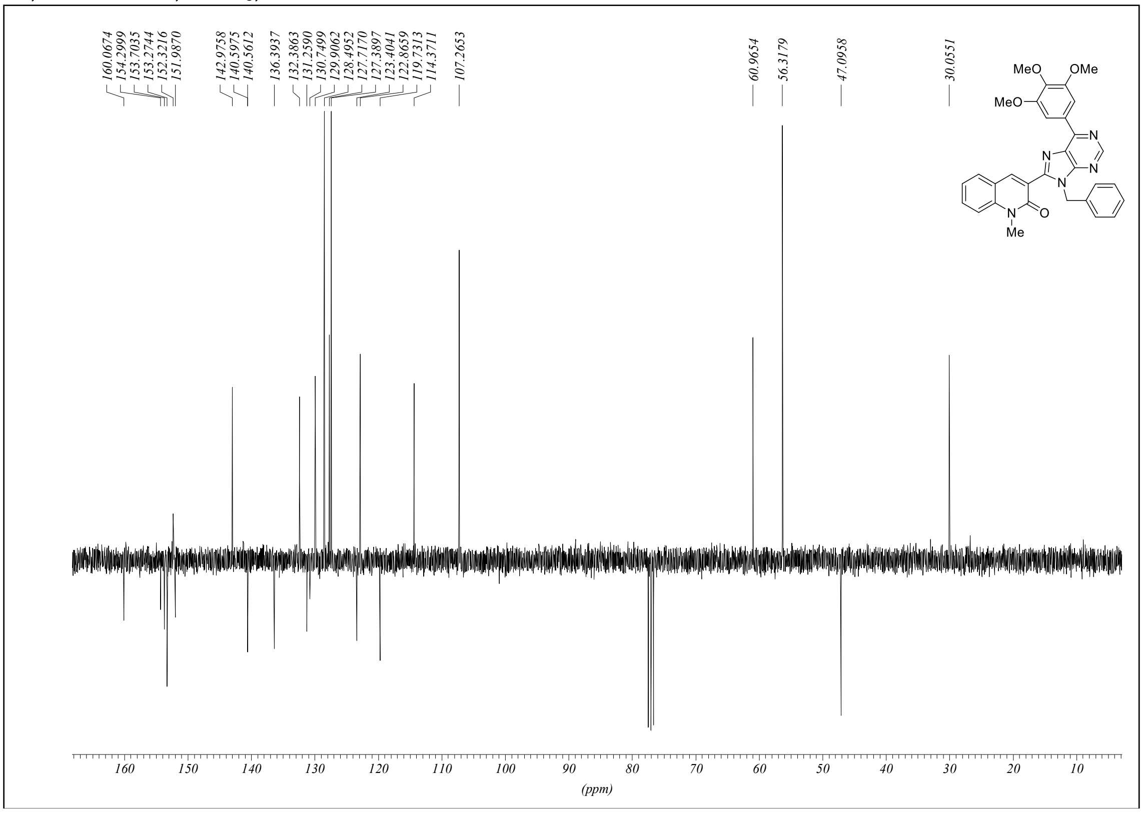

4d-HRMS

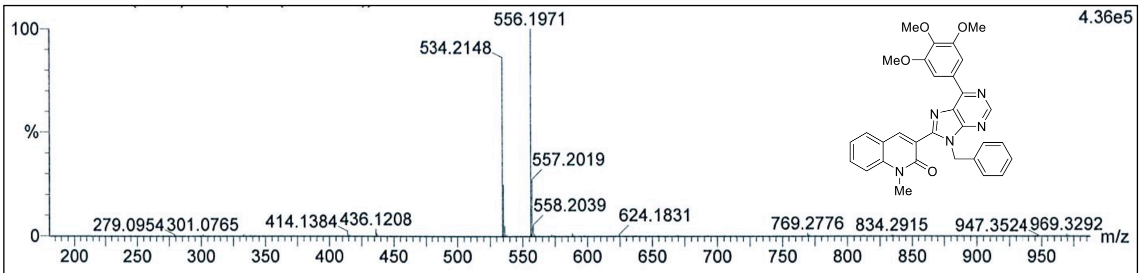

Mass	RA	Calc. Mass	mDa	PPM	DBE	i-FIT	Formula
Minimum:	50.00				-2.0		
Maximum:	100.00		200.0	5.0	100.0		
534.2148	86.25	534.2151	-0.3	-0.6	12.5	5963.4	C26 H33 N5 O4 Na S
		534.2141	0.7	1.3	20.5	3393.5	C31 H28 N5 O4
		534.2157	-0.9	-1.7	21.5	5556.6	C34 H29 N3 O2 Na
556.1971	100.00	556.1961	1.0	1.8	20.5	5420.2	C31 H27 N5 O4 Na
		556.1985	-1.4	-2.5	23.5	8290.3	C33 H26 N5 O4
		556.1994	-2.3	-4.1	15.5	10813.2	C28 H31 N5 O4 Na S

4e, ^1H NMR, $CDCl_3$, 300 MHz

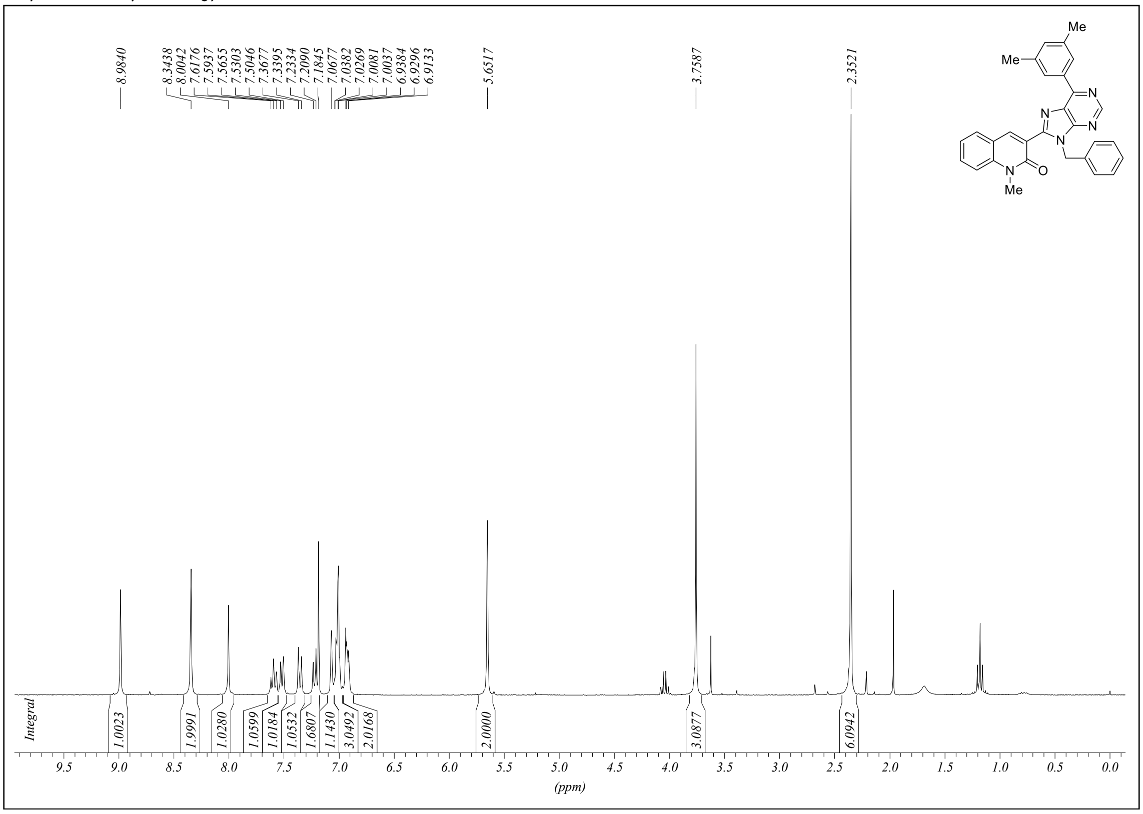

4e, JMOD NMR, $CDCl_3$, 75 MHz

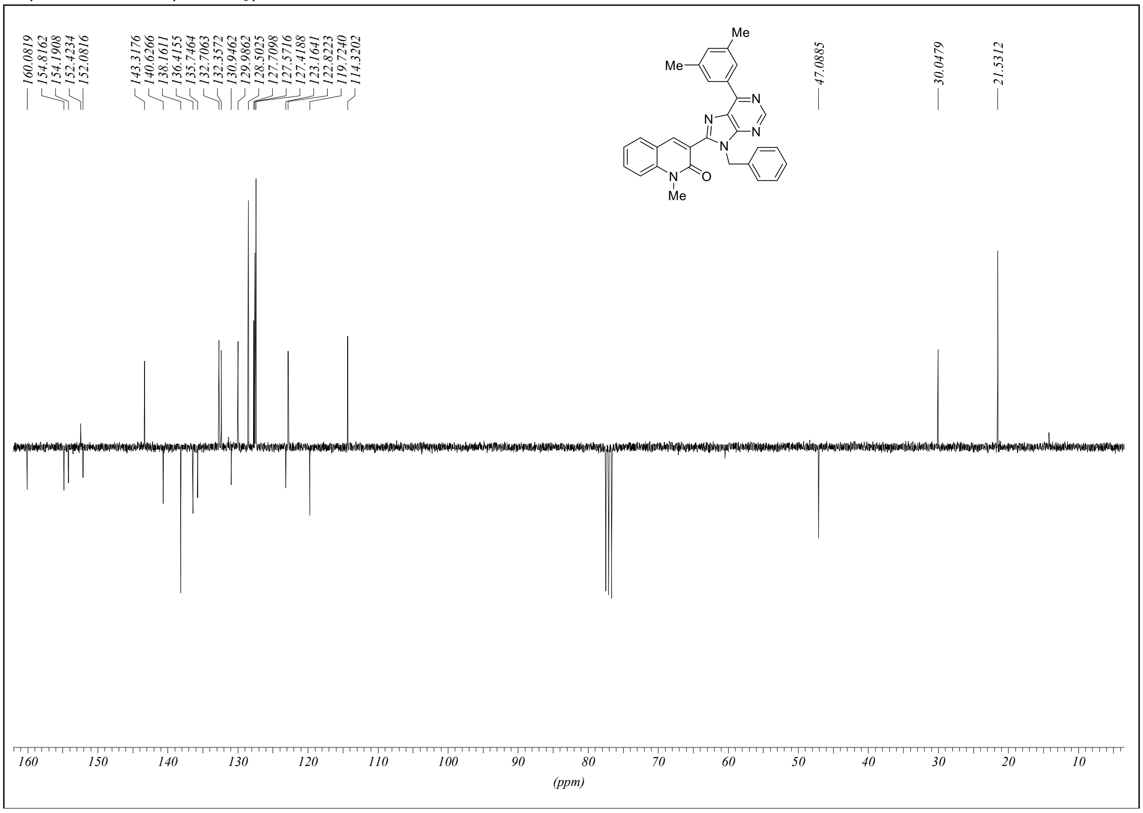

4e-HRMS

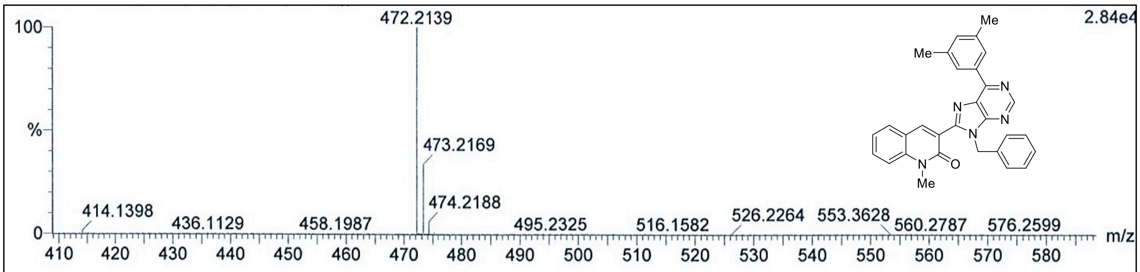

Minimum:	80.00				-2.0		
Maximum:	100.00		200.0	5.0	100.0		
Mass	RA	Calc. Mass	mDa	PPM	DBE	i-FIT	Formula
472.2139	100.00	472.2137	0.2	0.4	20.5	10.7	C30 H26 N5 O
		472.2147	-0.8	-1.7	12.5	266.1	C25 H31 N5 O Na S
		472.2124	1.5	3.2	15.5	4.0	C29 H30 N O5

4f, ^1H NMR, $CDCl_3$, 300 MHz

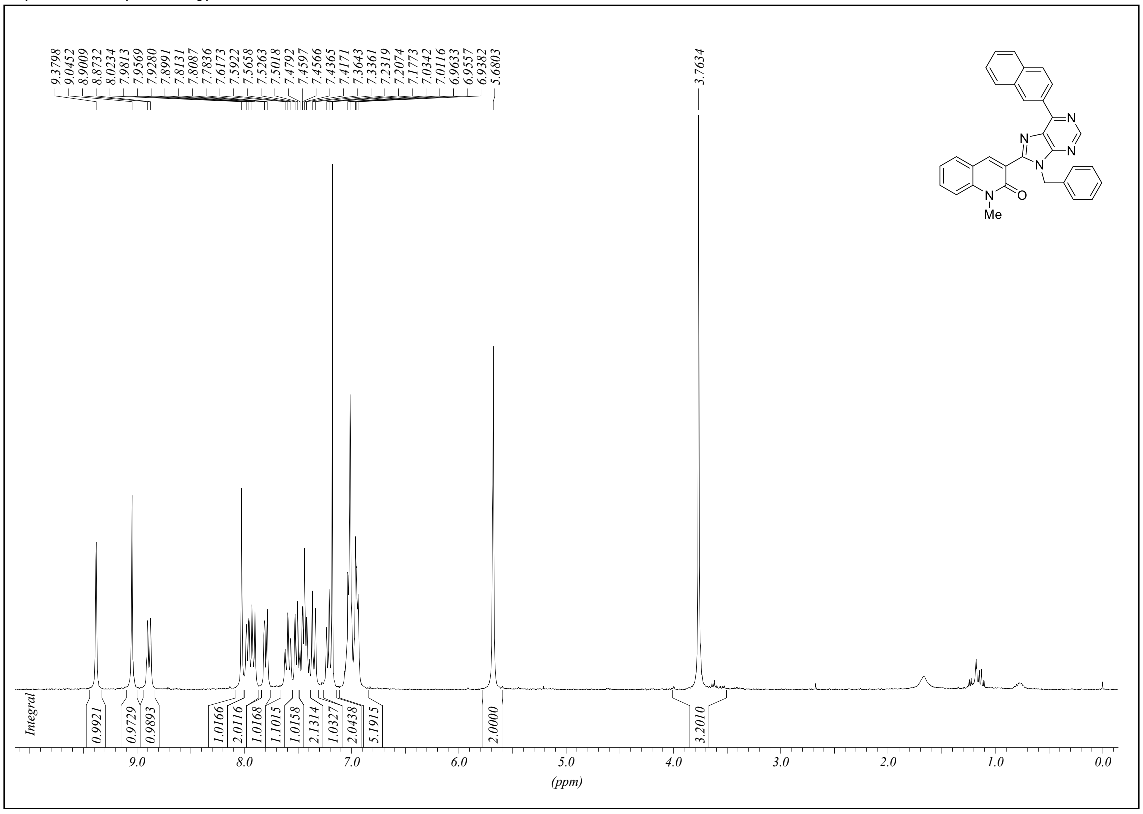

4f, JMOD-NMR, $CDCl_3$, 75 MHz

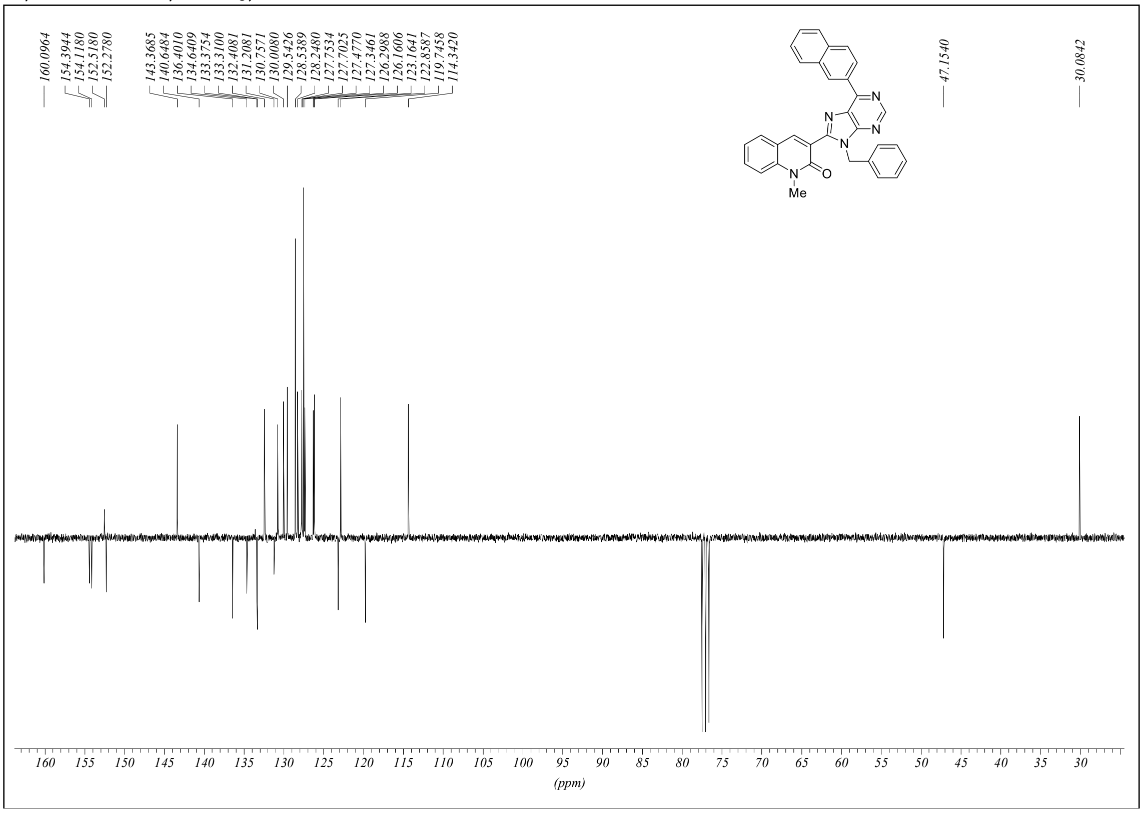

4f-HRMS

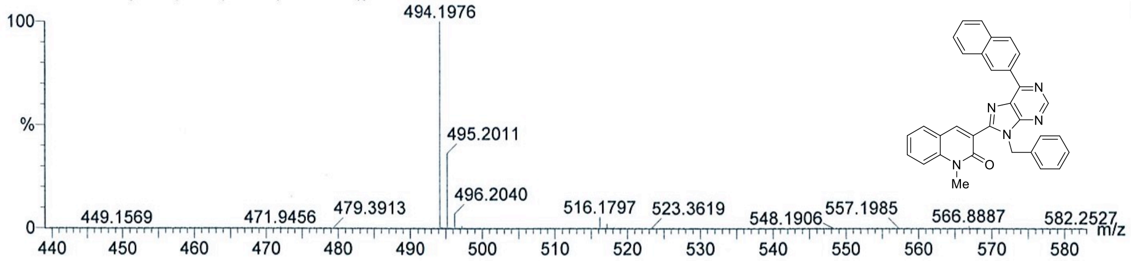

Minimum:	80.00				-2.0		
Maximum:	100.00		200.0	5.0	100.0		
Mass	RA	Calc. Mass	mDa	PPM	DBE	i-FIT	Formula
494.1976	100.00	494.1981	-0.5	-1.0	23.5	15.2	C32 H24 N5 O
		494.1967	0.9	1.8	18.5	5.9	C31 H28 N O5
		494.1957	1.9	3.8	20.5	11.2	C30 H25 N5 O Na

4g, ^{1}H NMR, $CDCl_3$, 300 MHz

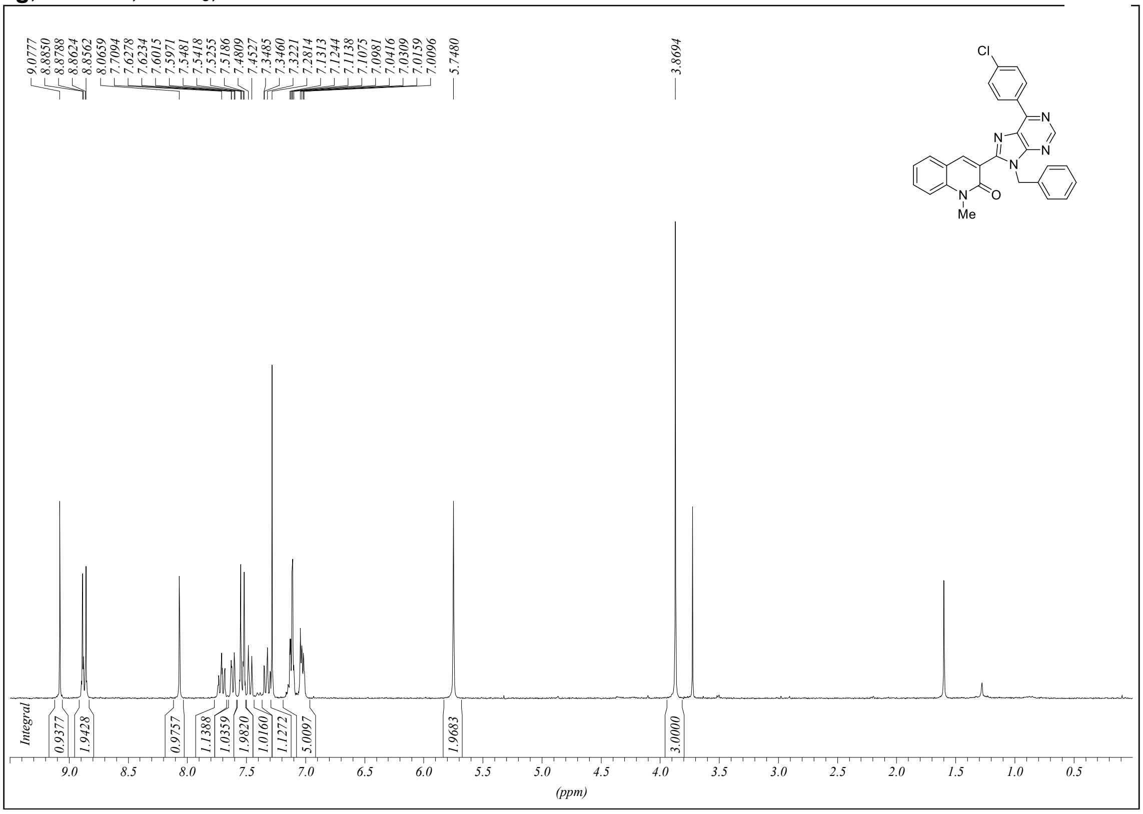

4g, ^{13}C NMR, $CDCl_3$, 75 MHz

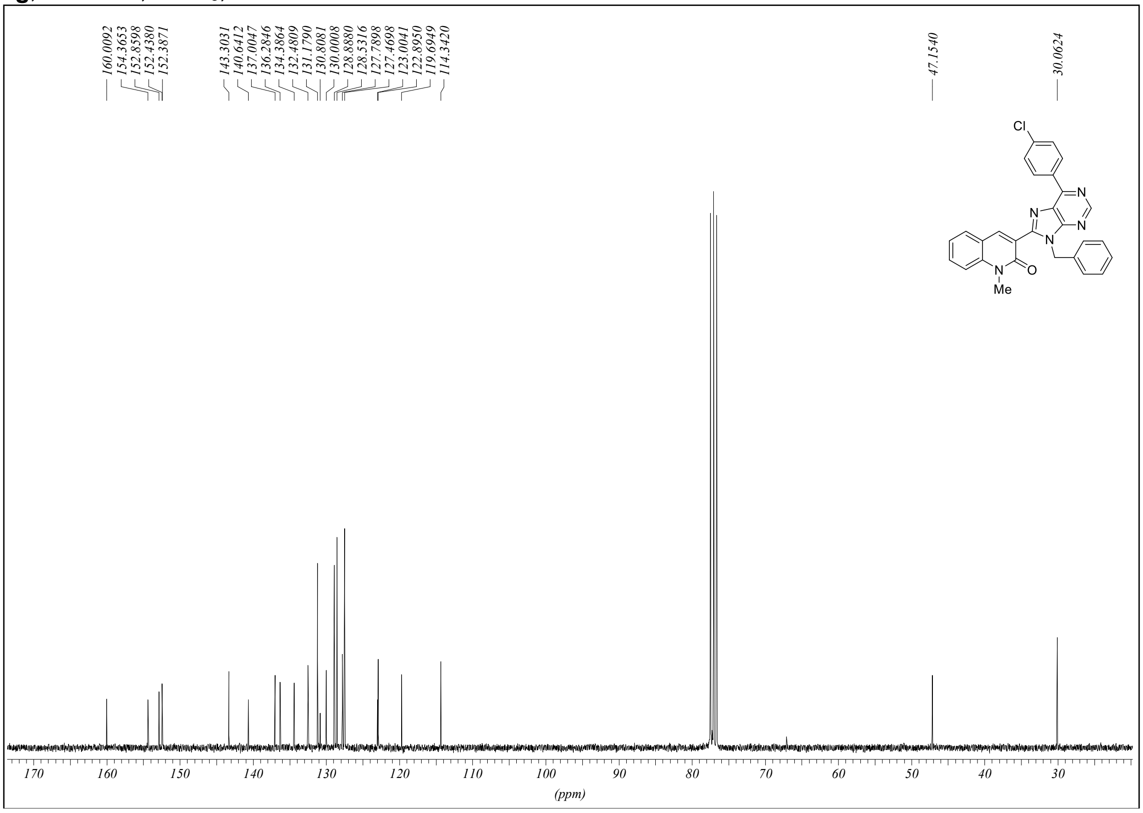

4g, HRMS

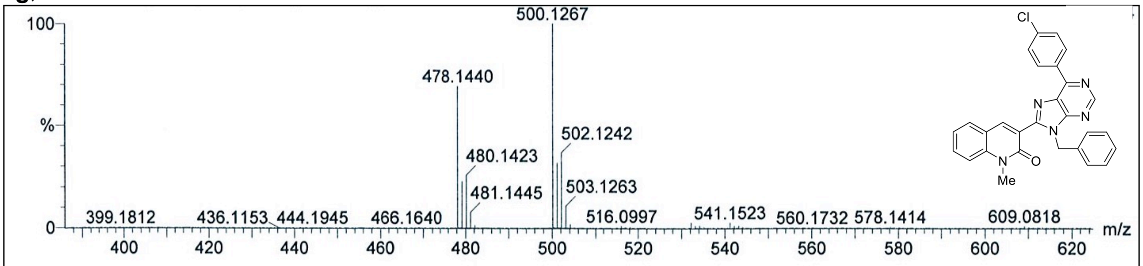

Minimum:	50.00				-2.0		
Maximum:	100.00		200.0	5.0	100.0		
Mass	RA	Calc. Mass	mDa	PPM	DBE	i-FIT	Formula
478.1440	69.35	478.1435	0.5	1.0	20.5	3.1	C28 H21 N5 O Cl
[M+H]+		478.1421	1.9	4.0	15.5	21.6	C27 H25 N O5 Cl
		478.1444	-0.4	-0.8	12.5	162.1	C23 H26 N5 O Na S Cl
500.1267	100.00	500.1254	1.3	2.6	20.5	19.5	C28 H20 N5 O Na Cl
[M+Na]+		500.1265	0.2	0.4	18.5	23.2	C29 H23 N O5 Cl
		500.1278	-1.1	-2.2	23.5	92.9	C30 H19 N5 O Cl

4h, ^{1}H NMR, $CDCl_3$, 300 MHz

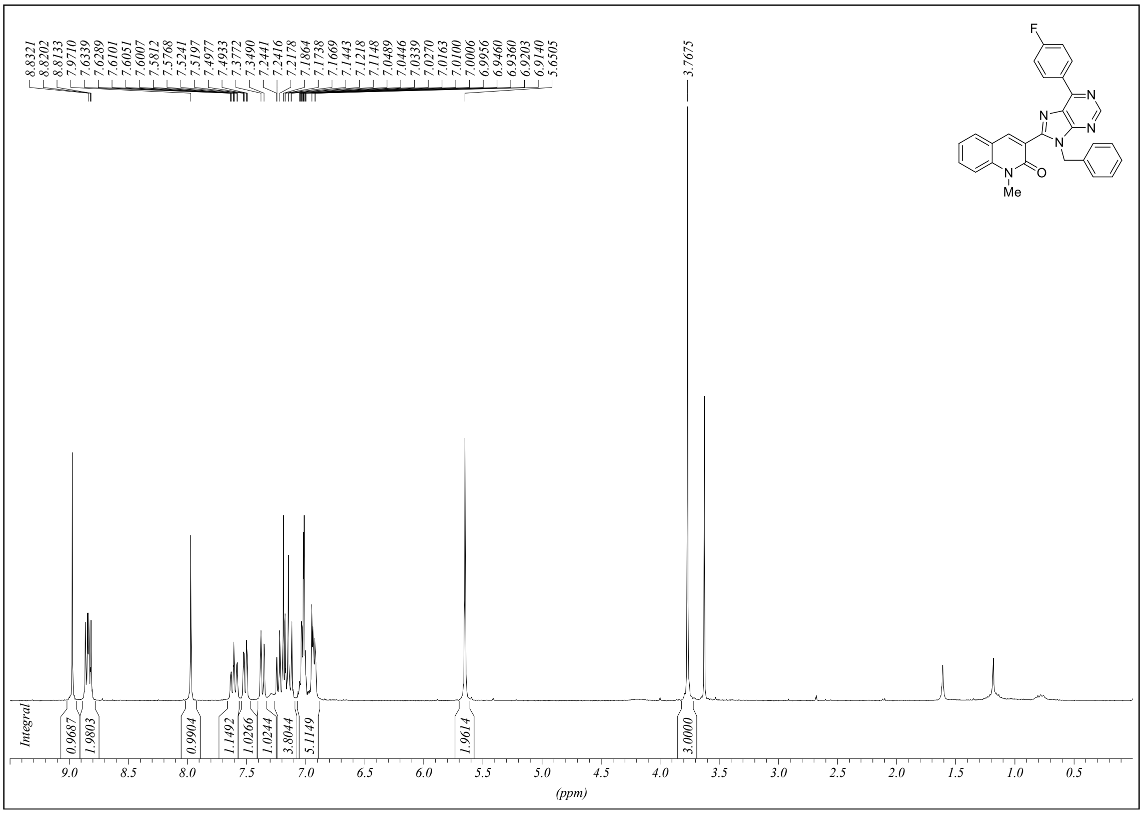

4h, JMOD-NMR, $CDCl_3$, 75 MHz

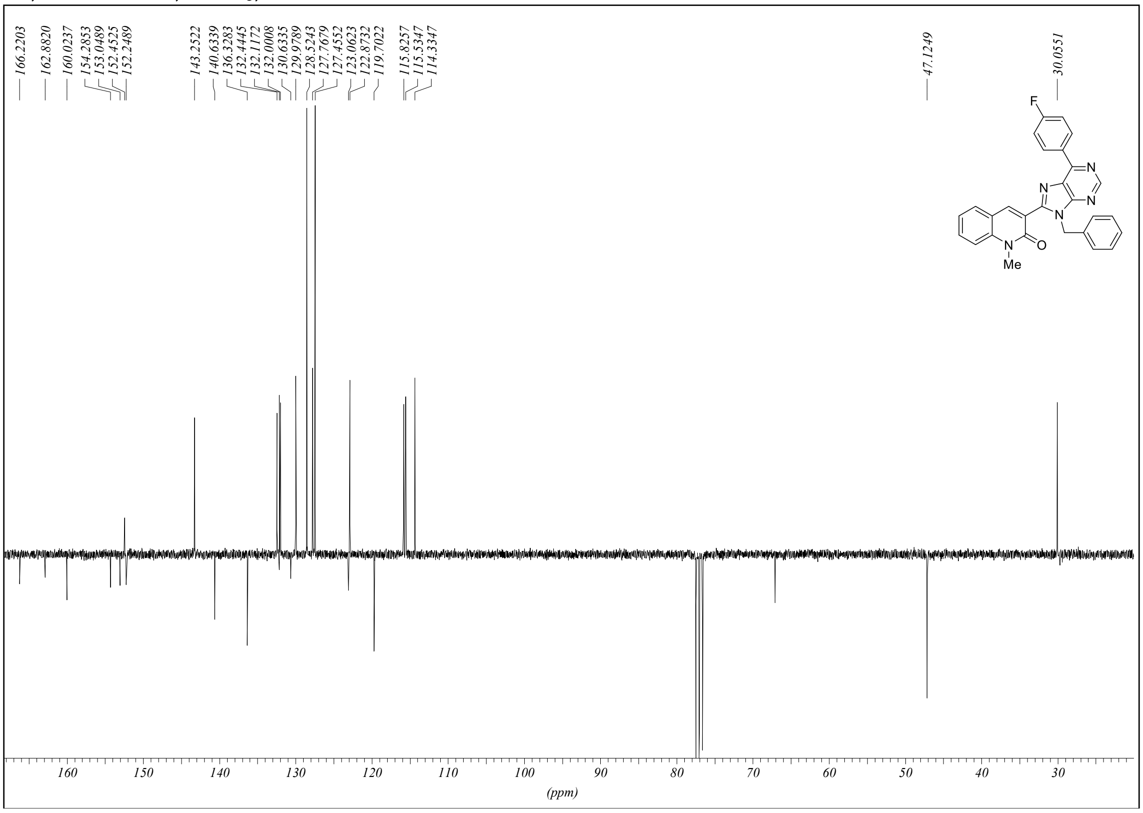

4h, HRMS

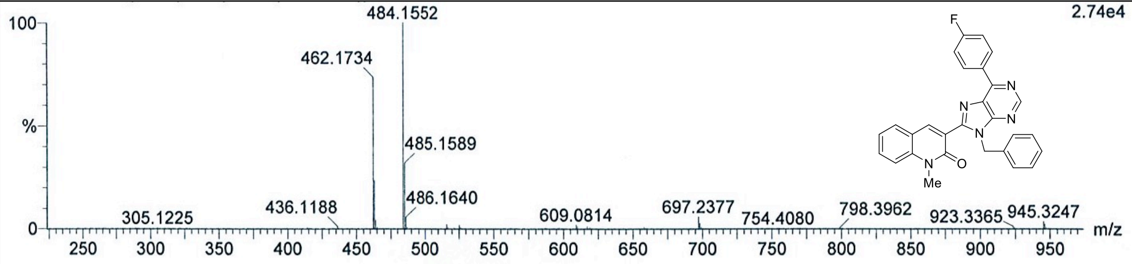

Minimum:	50.00				-2.0		
Maximum:	100.00		200.0	5.0	100.0		
Mass	RA	Calc. Mass	mDa	PPM	DBE	i-FIT	Formula
462.1734	73.35	462.1730	0.4	0.9	20.5	3.8	C28 H21 N5 O F
		462.1719	1.5	3.2	24.5	53.8	C31 H20 N5
		462.1717	1.7	3.7	15.5	4.3	C27 H25 N O5 F
484.1552	100.00	484.1550	0.2	0.4	20.5	7.4	C28 H20 N5 O F Na
		484.1560	-0.8	-1.7	18.5	16.8	C29 H23 N O5 F
		484.1536	1.6	3.3	15.5	4.3	C27 H24 N O5 F Na

5a, ^{1}H NMR, $CDCl_3$, 300 MHz

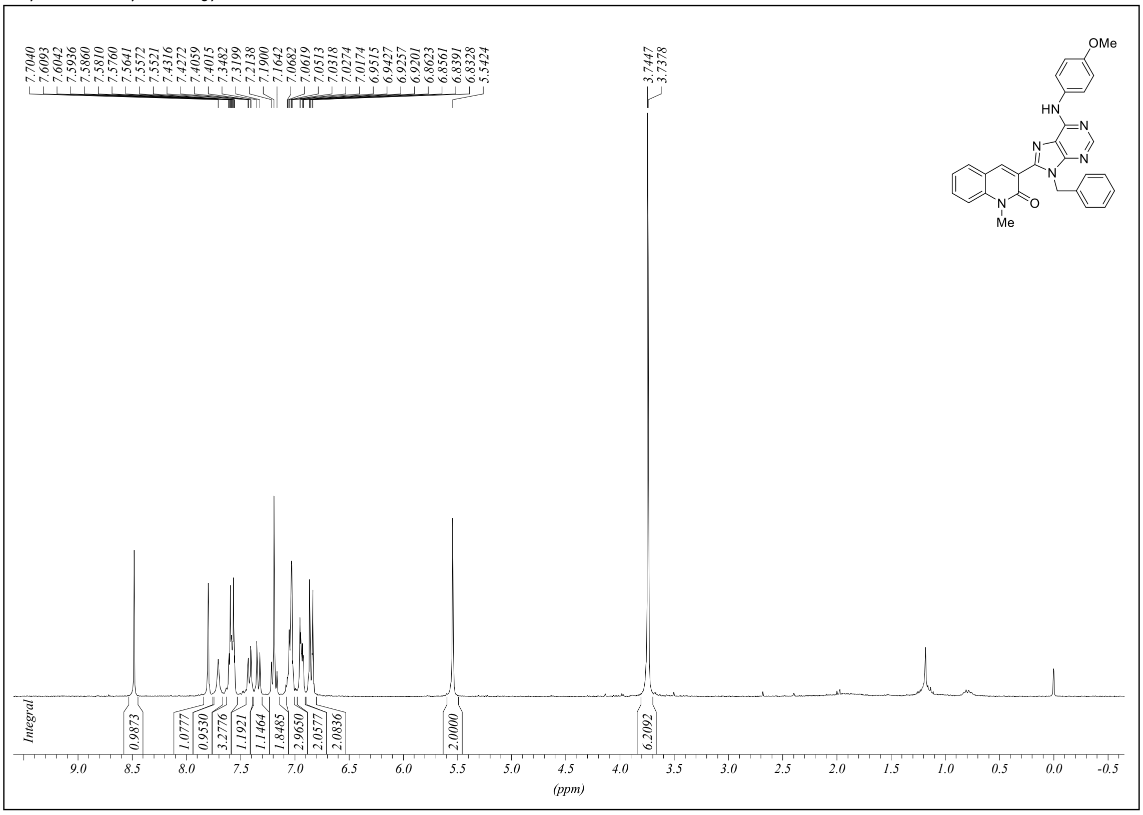

5a, ^{13}C-NMR, $CDCl_3$, 75 MHz

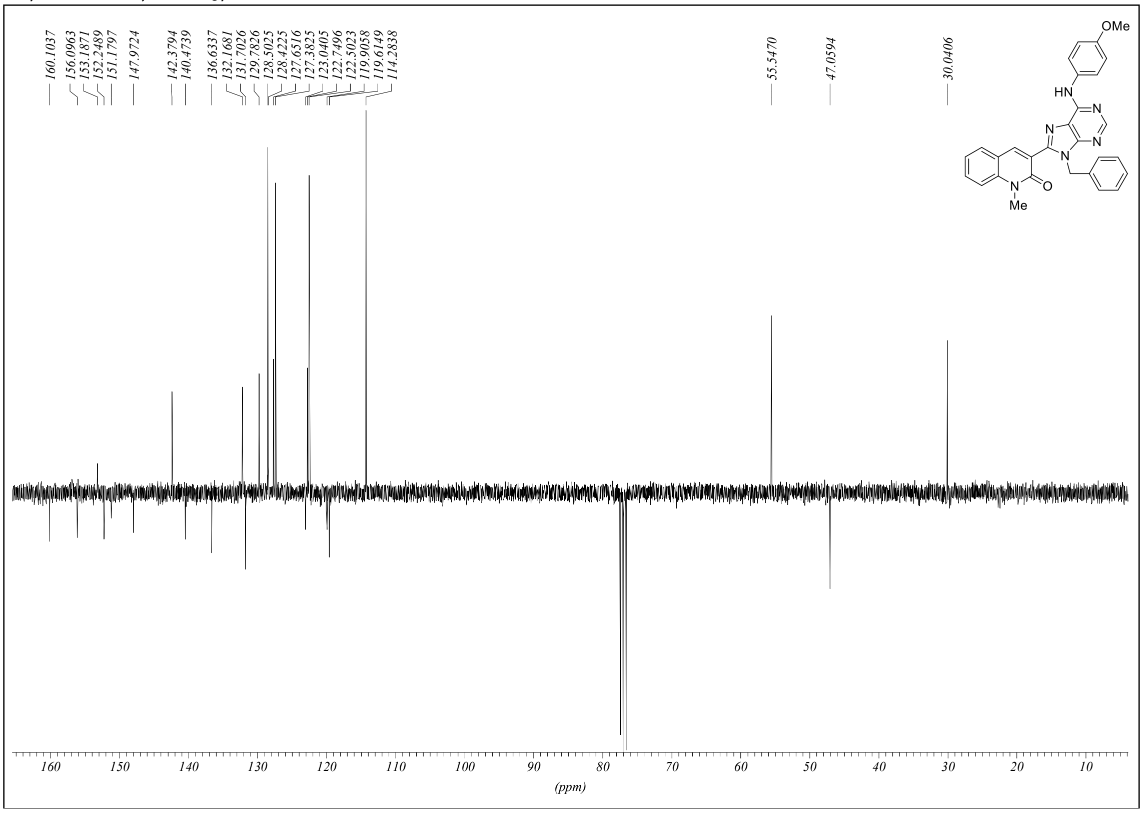

5a-HRMS

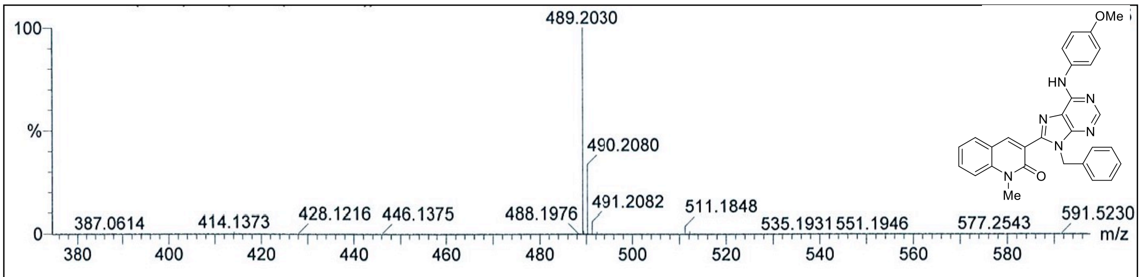

Minimum:	80.00				-2.0		
Maximum:	100.00		200.0	5.0	100.0		
Mass	RA	Calc. Mass	mDa	PPM	DBE	i-FIT	Formula
489.2030	100.00	489.2026	0.4	0.8	15.5	52.7	C28 H29 N2 O6
		489.2039	-0.9	-1.8	20.5	64.1	C29 H25 N6 O2
		489.2042	-1.2	-2.5	16.5	145.6	C31 H30 O4 Na

5b, ^{1}H NMR, $CDCl_3$, 300 MHz

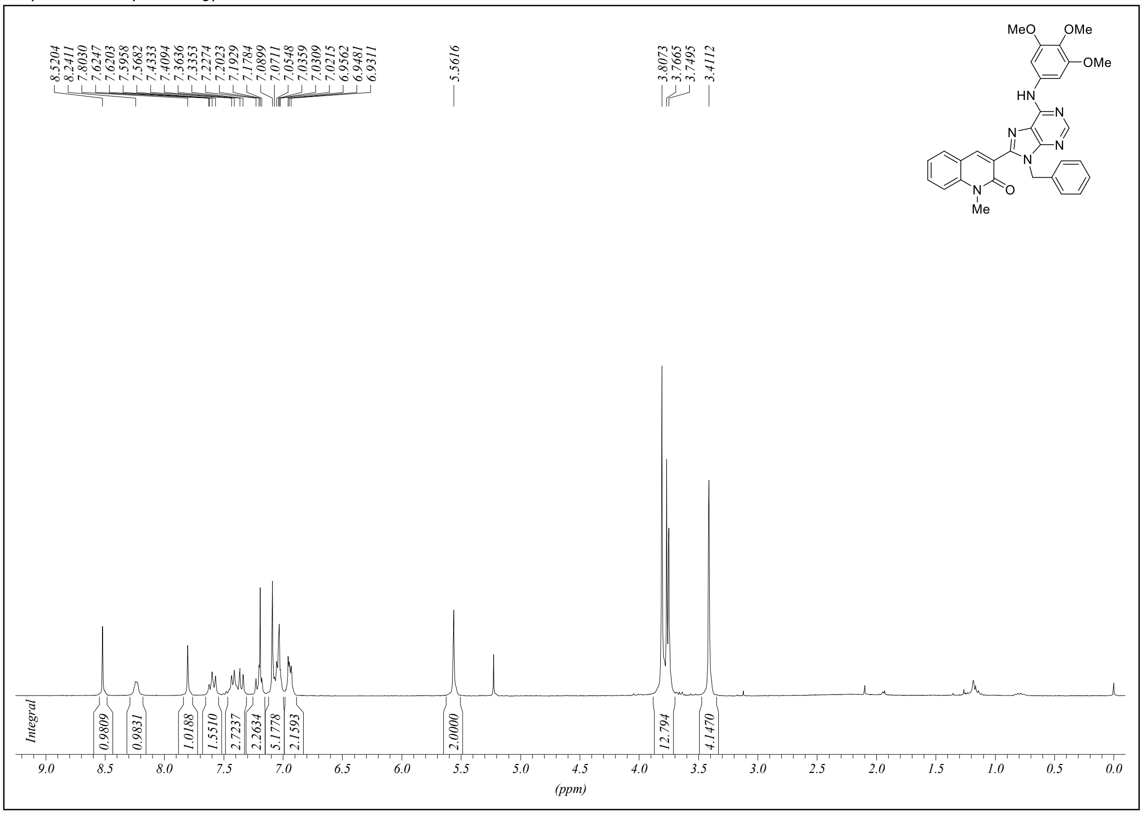

5b, JMOD-NMR, $CDCl_3$, 75 MHz

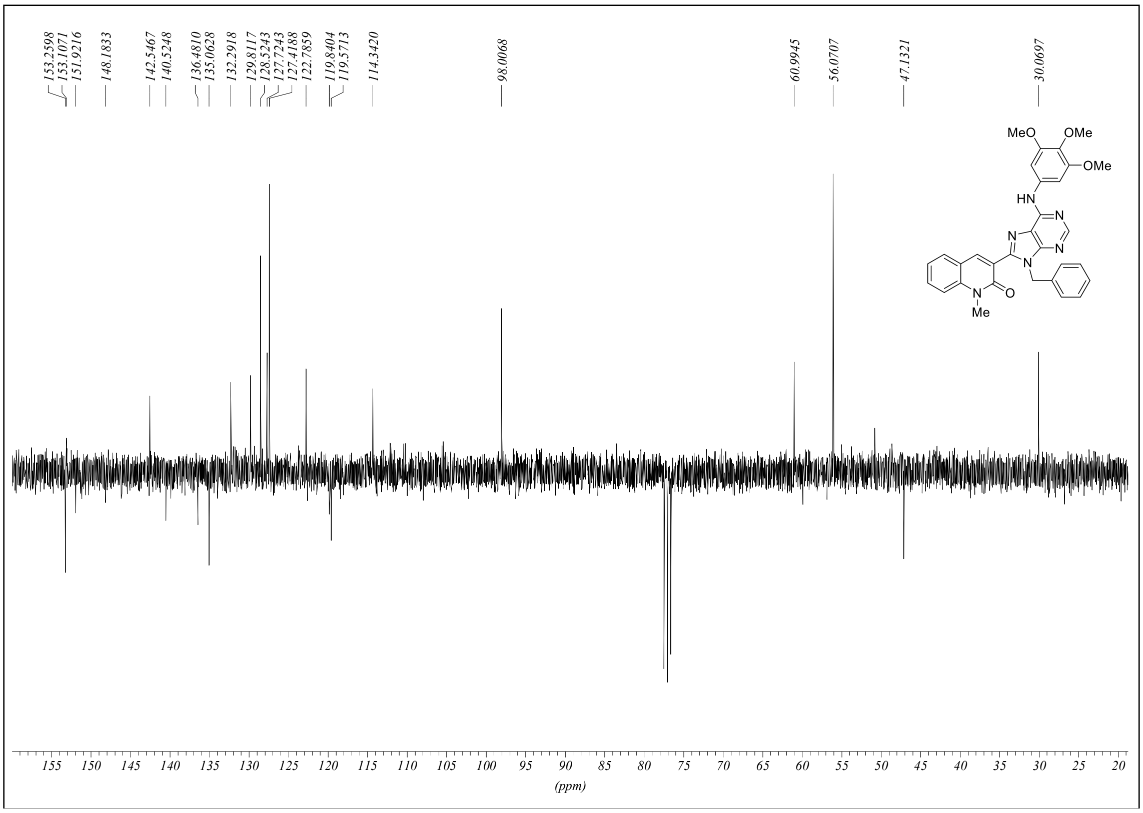

5b-HRMS

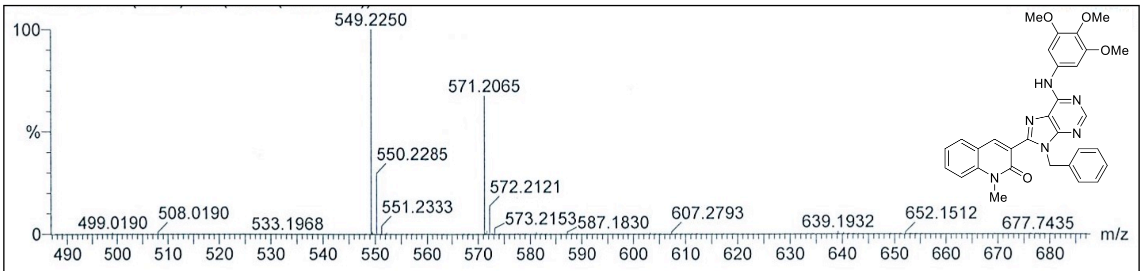

Minimum:	50.00				-2.0		
Maximum:	100.00		200.0	5.0	100.0		
Mass	RA	Calc. Mass	mDa	PPM	DBE	i-FIT	Formula
549.2250	100.00	549.2250	0.0	0.0	20.5	9313.1	C31 H29 N6 O4
		549.2253	-0.3	-0.5	16.5	10995.4	C33 H34 O6 Na
		549.2237	1.3	2.4	15.5	8121.4	C30 H33 N2 O8
571.2065	67.61	571.2062	0.3	0.5	31.5	37699.2	C44 H27 O
		571.2070	-0.5	-0.9	20.5	16020.4	C31 H28 N6 O4 Na
		571.2056	0.9	1.6	15.5	13257.7	C30 H32 N2 O8 Na
